# Identification of Host-Targeted Small Molecules That Restrict Intracellular *Mycobacterium tuberculosis* Growth

**DOI:** 10.1371/journal.ppat.1003946

**Published:** 2014-02-20

**Authors:** Sarah A. Stanley, Amy K. Barczak, Melanie R. Silvis, Samantha S. Luo, Kimberly Sogi, Martha Vokes, Mark-Anthony Bray, Anne E. Carpenter, Christopher B. Moore, Noman Siddiqi, Eric J. Rubin, Deborah T. Hung

**Affiliations:** 1 The Broad Institute of MIT and Harvard, Cambridge, Massachusetts, United States of America; 2 Division of Infectious Disease and Vaccinology, School of Public Health, University of California, Berkeley, Berkeley, California, United States of America; 3 Division of Infectious Disease, Massachusetts General Hospital, Boston, Massachusetts, United States of America; 4 Department of Molecular Biology and Center for Computational and Integrative Biology, Massachusetts General Hospital, Boston, Massachusetts, United States of America; 5 Department of Immunology and Infectious Diseases, Harvard School of Public Health, Boston, Massachusetts, United States of America; 6 Department of Microbiology and Immunobiology, Harvard Medical School, Boston, Massachusetts, United States of America; Weill Medical College of Cornell University, United States of America

## Abstract

*Mycobacterium tuberculosis* remains a significant threat to global health. Macrophages are the host cell for *M. tuberculosis* infection, and although bacteria are able to replicate intracellularly under certain conditions, it is also clear that macrophages are capable of killing *M. tuberculosis* if appropriately activated. The outcome of infection is determined at least in part by the host-pathogen interaction within the macrophage; however, we lack a complete understanding of which host pathways are critical for bacterial survival and replication. To add to our understanding of the molecular processes involved in intracellular infection, we performed a chemical screen using a high-content microscopic assay to identify small molecules that restrict mycobacterial growth in macrophages by targeting host functions and pathways. The identified host-targeted inhibitors restrict bacterial growth exclusively in the context of macrophage infection and predominantly fall into five categories: G-protein coupled receptor modulators, ion channel inhibitors, membrane transport proteins, anti-inflammatories, and kinase modulators. We found that fluoxetine, a selective serotonin reuptake inhibitor, enhances secretion of pro-inflammatory cytokine TNF-α and induces autophagy in infected macrophages, and gefitinib, an inhibitor of the Epidermal Growth Factor Receptor (EGFR), also activates autophagy and restricts growth. We demonstrate that during infection signaling through EGFR activates a p38 MAPK signaling pathway that prevents macrophages from effectively responding to infection. Inhibition of this pathway using gefitinib during *in vivo* infection reduces growth of *M. tuberculosis* in the lungs of infected mice. Our results support the concept that screening for inhibitors using intracellular models results in the identification of tool compounds for probing pathways during *in vivo* infection and may also result in the identification of new anti-tuberculosis agents that work by modulating host pathways. Given the existing experience with some of our identified compounds for other therapeutic indications, further clinically-directed study of these compounds is merited.

## Author Summary

Infection with the bacterial pathogen *Mycobacterium tuberculosis* causes the disease tuberculosis (TB) that imposes significant worldwide morbidity and mortality. Approximately 2 billion people are infected with *M. tuberculosis*, and almost 1.5 million people die annually from TB. With increasing drug resistance and few novel drug candidates, our inability to effectively treat all infected individuals necessitates a deeper understanding of the host-pathogen interface to facilitate new approaches to treatment. In addition, the current anti-tuberculosis regimen requires months of strict compliance to clear infection; targeting host immune function could play a strategic role in reducing the duration and complexity of treatment while effectively treating drug-resistant strains. Here we use a microscopy-based screen to identify molecules that target host pathways and inhibit the growth of *M. tuberculosis* in macrophages. We identified several host pathways not previously implicated in tuberculosis. The identified inhibitors prevent growth either by blocking host pathways exploited by *M. tuberculosis* for virulence, or by activating immune responses that target intracellular bacteria. Fluoxetine, used clinically for treating depression, induces autophagy and enhances production of TNF-α. Similarly, gefitinib, used clinically for treating cancer, inhibits *M. tuberculosis* growth in macrophages. Importantly, gefitinib treatment reduces bacterial replication in the lungs of *M. tuberculosis*-infected mice.

## Introduction

Tuberculosis continues to be a cause of significant morbidity and mortality world-wide due to numerous factors, including the rise of drug resistance and the absence of an effective vaccine. The challenge of adherence to long treatment regimens and the limited number of effective therapeutics drive the need for innovative therapeutic strategies that are predicated on a better understanding of the biology of infection. For example, the fact that the majority of people infected with the bacillus never develop active disease suggests that human immunity is actually quite effective at controlling *M. tuberculosis*; a novel, potentially more effective therapeutic strategy could emerge if we were able to understand and leverage the basis for this control.

The cellular interaction between *M. tuberculosis* and macrophages is crucial for determining the outcome of infection. Early in infection, macrophage microbicidal mechanisms actively work to try to clear the bacteria; however, macrophage responses that are adequate to kill other bacterial pathogens often fail to clear *M. tuberculosis*. In a majority of individuals, the activation of macrophages by IFN-γ can result in control but not sterilization of infection, instead driving it into a latent state. During latency, the macrophage can be a niche where the bacteria are protected from assaults by the immune system and antibiotic therapy, thus facilitating their persistence and ultimate dissemination.

Recent studies have uncovered a number of processes that are important to tubercular infection. The ability of *M. tuberculosis* to arrest the normal progress of phagosome maturation is critical for its survival in macrophages [1]; however, the molecular mechanisms on both the pathogen and host sides that account for this arrest are unclear. For example, while calcium signaling in macrophages appears to be important in this process, the nature of the calcium signal and the mechanisms by which *M. tuberculosis* actively affects calcium signaling are debated [2], [3]. In addition to phagosome maturation arrest, *M. tuberculosis* may actively suppress many other macrophage innate immune responses. For example, virulent strains of *M. tuberculosis* actively prevent apoptosis of infected macrophages, thus preventing bacterial killing by macrophage efferocytosis and avoiding activation of T-cells through cross-presentation of antigens by dendritic cells [4], [5], [6]. *M. tuberculosis* may also actively prevent activation of the inflammasome and induction of autophagy [7], [8]. In addition to subversion of immune responses, *M. tuberculosis* manipulates the host microenvironment in order to acquire nutrients to promote its own survival. For example, virulent mycobacteria are able to induce the development of intracellular lipid bodies which fuse with *M. tuberculosis* containing phagosomes and provide a critical source of carbon [9].

Although we have some insight into the pathways that are important for *M. tuberculosis* infection of macrophages, our current understanding of the mechanisms that determine whether the macrophage controls bacterial infection or succumbs to its virulence is incomplete. In order to obtain greater insight into host factors involved in *M. tuberculosis* infection, unbiased screening using RNAi or small molecules targeting host proteins have recently been performed. Two published RNAi screens, one genome-wide and one focused on kinases and phosphatases, identified mammalian proteins that are candidate regulators of *M. tuberculosis* infection [10], [11]. To provide a functional context for the identified regulators, the authors constructed a signaling network by integrating the RNAi screening data with data from transcriptional profiling. Over half of identified genes were found to be negative regulators of autophagy, affirming the importance of this pathway for host defense against *M. tuberculosis* [10]. In addition to regulators of autophagy, the networks implicated were enriched for modules that govern metabolism and signal transduction, with many of these modules centered around the serine/threonine kinase AKT.

Kinases are central to mammalian signaling pathways. AKT/PKB is a key modulator of cellular processes such as growth and proliferation, glucose metabolism, apoptosis, and autophagy. AKT is specifically activated during *Salmonella* infection of host cells by the bacterial effector SopB and promotes bacterial survival by prevention of phago-lysosome fusion [12]. Treatment of *M. tuberculosis* macrophages with the AKT and PKA inhibitor H-89 also results in inhibition of bacterial growth. However, in contrast to *Salmonella* infection, the role of AKT is unknown in *M. tuberculosis* infection [12]. Importantly, although AKT was identified in the network that emerged from the genome-wide RNAi screen of *M. tuberculosis* infected THP-1 macrophages, the kinase itself was neither identified in the primary genome-wide screen nor in a more directed kinase/phosphatase screen conducted by the same group [11].

Of note, in the RNAi screens that have been reported, the siRNAs used to decrease host factor expression were added only after *M. tuberculosis* had already entered and adapted to the macrophage microenvironment; thus, these screens were not designed to identify factors that are crucial for the earliest events in the host-pathogen interaction. Effective silencing of gene expression using transfection of siRNA is in part dependent on the half-life of the targeted protein and occurs on the timescale of hours to days after transfection. In contrast, a chemical biological approach has some advantages over RNAi with regard to studying early events. The rapid binding of small molecules to proteins facilitates probing the early period immediately after phagocytosis. Because this period is accompanied by the most significant transcriptional responses on the part of both the bacterium and the macrophage [13], events during this time frame are likely important determinants of the outcome of infection. Further, in general, unlike genetic approaches which can target only one isoform or homologue at a time, small molecules can inhibit multiple, closely related isoforms of the same target, thus facilitating the identification of host activities performed by two or three closely related targets with redundant function. In addition, unlike RNAi, small molecules can inhibit enzymatic function without disrupting larger complexes with subsequent pleiotropic effects, or can inhibit a specific function of a protein while leaving other functions intact [14]. Thus small molecule based screens can provide a valuable complement to existing datasets obtained using siRNA based knockdown.

Three recently conducted screens were designed to identify small molecules that disrupt *M. tuberculosis* replication in macrophages. The first screen used a high-content imaging approach to identify compounds that directly target bacterial processes during macrophage growth [15]. A subsequent study by the same group and using the same screening approach identified an inhibitor of *M. tuberculosis* cytochrome bc_1_ [16]. More recently, a group reported a screen in which microscopy was used to identify host-targeted inhibitors that prevent replication of *Mycobacterium bovis* BCG in macrophages. This study characterized existing neurotropic drugs that diminish replication of *M. tuberculosis* in infected macrophages by inducing autophagy or altering endosomal trafficking, however the targets of these drugs and their modes of action have yet to be elucidated [17]. We performed a complementary screen of a library of known bioactive small molecules to identify inhibitors of *M. tuberculosis* replication in macrophages that are biased towards disrupting host functions. Our goal was to identify small molecules that specifically target host proteins to gain insight into host functions required for controlling *M. tuberculosis* infection. To focus on early events crucial for the adaptation of the macrophage for bacterial survival but not on uptake itself, we added molecules to infected macrophages immediately after phagocytosis. From this screen, we identified several classes of host-targeted compounds that limit the ability of *M. tuberculosis* to proliferate in macrophages, including kinase inhibitors, G-protein coupled receptor modulators, and ion channel inhibitors. Our results are complementary to previously reported screens, expanding our knowledge of host pathways that are crucial determinants of infection. Based on the bioactive molecules identified in our screen, we determined that the SSRI fluoxetine inhibits *M. tuberculosis* growth in macrophages, induces enhanced expression of TNF-α, and enhances autophagy. We additionally demonstrated a new role for the tyrosine kinase EGFR during *M. tuberculosis* infection. Importantly, we showed that the clinically used EGFR inhibitor gefitinib has efficacy for preventing bacterial replication in both infected macrophages and mice, suggesting that EGFR is relevant *in vivo*. The inhibitors we have identified will be important tools for studying the host-pathogen interaction and for testing the importance of host pathways during *in vivo* infection in animal models.

## Results

### Development of a high-throughput, high-content imaging assay to identify compounds that inhibit *M. tuberculosis* growth in macrophages

For identification of small molecules that inhibit *M. tuberculosis* replication in macrophages, after significant assay testing (see Methods S1) we designed a microscopy-based assay with simultaneous imaging of macrophages and mycobacteria that provides the sensitivity to detect modest growth inhibition, easily identifies compounds with significant macrophage toxicity, and can be largely automated to allow for the rapid testing of thousands of compounds (for assay design and development details, see Methods S1). We used this high-content imaging assay to monitor the growth of GFP-expressing *M. tuberculosis* in infected macrophages.

In designing the assay, we sought to establish growth conditions that would allow for robust and reproducible replication of *M. tuberculosis* in macrophages and a readout that would accurately reflect bacterial number. Macrophages were infected with *M. tuberculosis* constitutively expressing GFP (H37Rv-GFP [18]), and at various time-points after infection, the cells were fixed and stained with DAPI to allow for enumeration of macrophages by microscopy. Because intracellular mycobacteria grow in clumps rather than as discrete, easily quantifiable spots, we utilized the open-source image analysis software CellProfiler, which can simultaneously determine and integrate multiple parameters from our images [19] to determine an optimal visual parameter that correlates with bacterial number. We found that GFP pixel intensity integrated across the field and normalized to macrophage number best represented growth while also accounting for differences in surviving macrophage number, and most accurately reflected bacterial census enumerated by plating for colony forming units (CFU).

Using this parameter, we tested growth of H37Rv-GFP in a number of cell lines including THP-1 cells, RAW 264.7, and J774A.1 cells, and found that J774 murine macrophages allowed for the most consistent and robust growth, with a bacterial doubling time equivalent to that measured in axenic culture. The optimal time-point for measuring growth was 3 days post infection, when intracellular mycobacterial growth was most homogeneous and little apparent macrophage death was observed.

**Figure 1:**
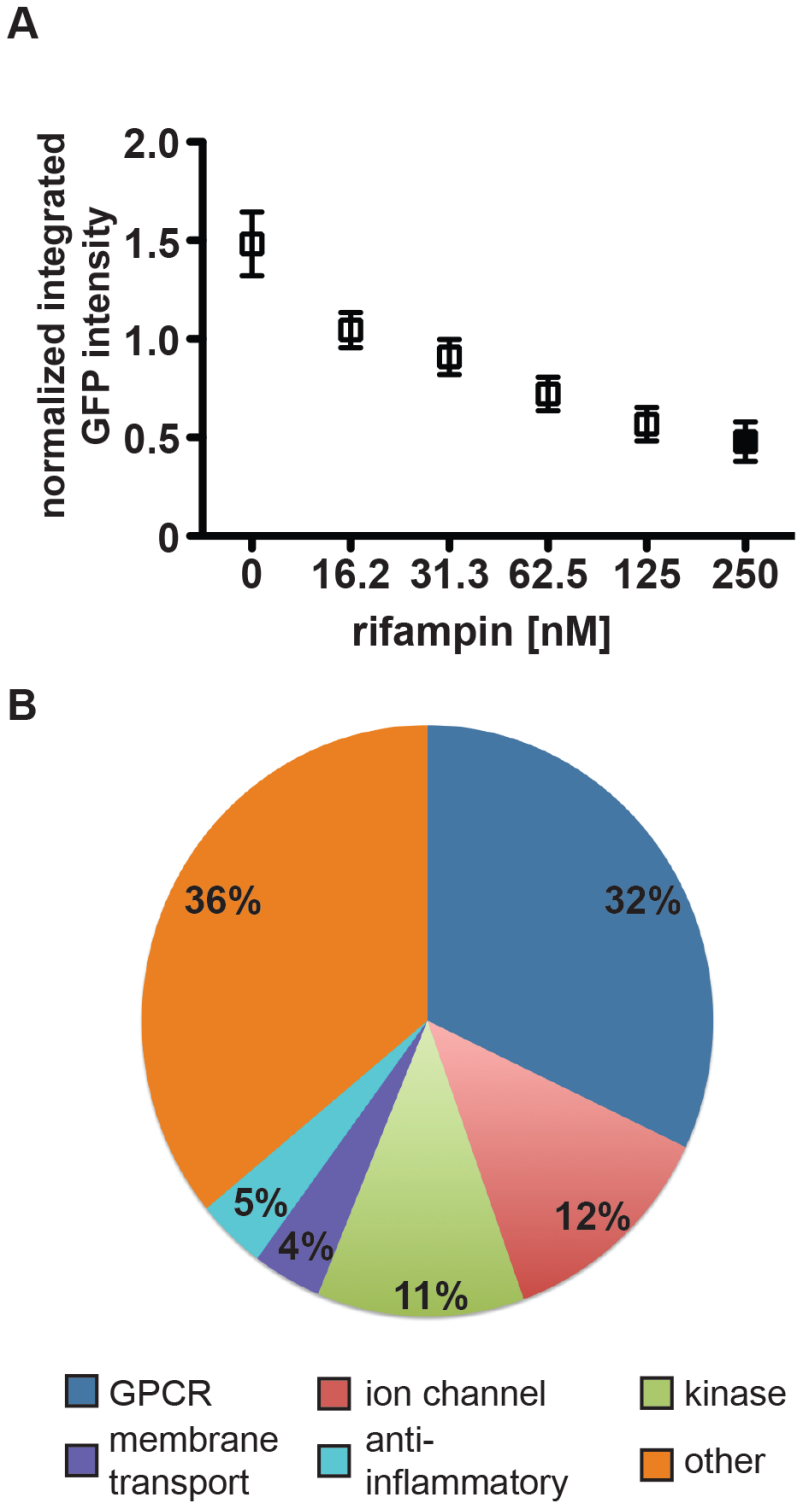
High-content imaging assay development and categorization of screen hits. (A) J774 cells in 96-well dishes were infected with GFP-expressing H37Rv at an MOI of 1∶1. Cells were then treated with rifampin at the indicated concentrations. Day 3 after infection, cells were fixed, stained with DAPI, and imaged for both DAPI and GFP. Images were analyzed using CellProfiler to determine the mean integrated GFP intensity across each well normalized to macrophage number. The graph represents one of four independent experiments with data represented as an average +/− SEM for each condition for 84 wells. (B) Of 133 unique hits in the screen, 41 were agonists or antagonists at G-protein coupled receptors, 16 were inhibitors or activators of ion channels, 15 were kinase activators or inhibitors, 5 were membrane transport modulators, 7 were anti-inflammatory agents, and 46 did not fall into one of the above major categories.

We next assessed the heterogeneity of mycobacterial growth and ability of the assay to reproducibly detect growth inhibition across large numbers of wells in 96-well format. Using a gradient of concentrations of rifampicin to model varying degrees of growth inhibition, we demonstrated that the assay was able to distinguish growth inhibition of 50% inhibition or less (Figure 1A, Figure S1). With the highest dose of rifampicin we consistently obtained Z′-factors of 0.4–0.5, which is borderline in robustness for a high-throughput screen. However, because bacterial growth based on imaging across the wells did not follow a Gaussian distribution, standard statistical methods to assess high-throughput assay quality, such as Z′-factor, that assume a normal distribution for high-throughput assays may not accurately assess the robustness of the assay. We therefore used a bootstrap Monte Carlo analysis as another means to assess the quality of the assay [20], [21] and found that we were able to consistently distinguish 50% inhibition of growth as established with our positive controls with a predicted false negative rate of 0.2% and a false positive rate of 0.13%. We found these rates to be acceptable, allowing us to progress to high-throughput screening. Ultimately we compared the list of hits obtained using either composite z-scores or p-values obtained using the Monte Carlo bootstrap analysis (both metrics included in Table S1); we found that the list of hits was essentially the same using both metrics.

### Screening a library of bioactive small molecules and screen hits

We used the assay to screen a library of 1920 small molecules from the Broad Institute bioactives collection and an additional 159 kinase inhibitors obtained from Nathanael Gray at the Dana Farber Cancer Institute (Table S1, Figure S2). One advantage of screening a library of known bioactives is that compound annotation can provide initial suggestions about mechanisms of action, thus facilitating the considerable challenge of target identification from potential host and bacterial targets. Additionally, the bioactives library is enriched for molecules that target mammalian proteins, thus allowing for elucidation of novel host/pathogen biology by focusing on macrophage functions.

In a 96 well format, ∼3000 macrophages were plated in each well and infected with *M. tuberculosis* strain H37Rv-GFP at a multiplicity of infection (MOI) of 1∶1 for 4 hours, after which time extracellular bacteria were removed by washing. To facilitate identification of compounds that restrict intracellular *M. tuberculosis* growth rather than inhibit uptake, the compound library was added after phagocytosis. The average final concentration of small molecules was 5 µM. After three days, cells were washed, fixed, stained with DAPI, and imaged with a 4× objective lens on an ImageXpress Micro high-throughput microscope (Molecular Devices).

We analyzed our data using both a bootstrap Monte Carlo analysis and composite z-score and were reassured that both methods identified the same small molecule hits. (The z-score cutoff was <−1.5; the p-value cutoff was <0.025.) From the primary screen we identified a total of 164 unique small molecules that resulted in statistically significant growth inhibition when compared to control wells. We discarded compounds with significant macrophage toxicity (<50% macrophage survival relative to controls), known antibiotics, and compounds identified to have significant activity against *M. tuberculosis* grown in axenic culture in a parallel screen [18]. After applying these filters, we were left with 133 unique non-antibiotic compounds (Table S1) that restrict growth of *M. tuberculosis* in macrophages. Based on known annotation, most identified molecules fall into 5 broad categories: G-protein coupled receptor (GPCR) modulators, ion channel modulators, membrane transport protein-acting compounds, anti-inflammatories, and kinase modulators (Figure 1B). Categorizing compounds based on known annotations raises the caveat that activity in this assay may not necessarily be related to their annotated activity but rather to another “off-target” effect. However, we compared the relative representation of activity classes from active molecules with the library that was screened and found a slight over-representation of GPCR modulators, ion channel modulators, and membrane transport protein-acting compounds, and similar representation among anti-inflammatory compounds and kinase modulators. The specific categories identified and relative proportions of hits in each category reflect both underlying biology and representation of these categories in the screened library. To assess the success of the screen, we selected and repurchased a group of 22 compounds with a range of composite z-scores (−4 to −1.5) for retesting. Importantly, our retest rate from these compounds was 90%.

### Confirmation of screening candidates by select compound retesting

**Figure 2:**
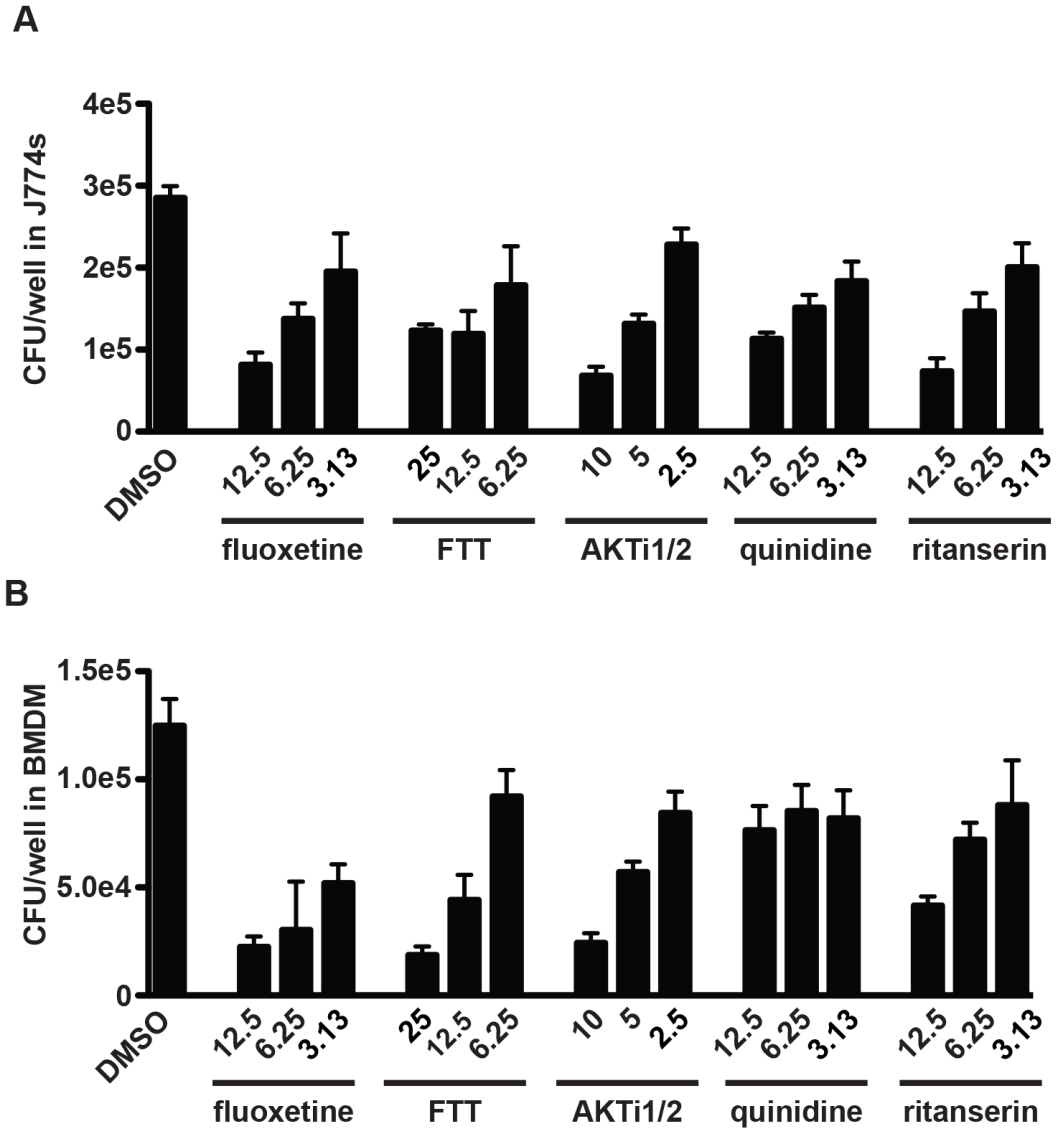
Selected screen hits have dose-dependent activity in J774 cells and bone-marrow derived macrophages. Selected hit compounds were retested at varying doses in (A) J774 murine macrophages and (B) mouse bone marrow-derived macrophages (BMDM). Cells were infected with *M. tuberculosis* strain H37Rv at an MOI of 1∶1 and treated with each compound at the indicated doses (µM) after a 4 h phagocytosis period. At day 3 (J774) or day 5 (BMDM) after infection, cells were washed, lysed, and plated for CFU. Each column represents the mean and standard deviation of four biological replicates. Each experiment was repeated three times and a representative experiment is shown. With the exception of fluoxetine at 6.25 µM, all p-values were <0.05 for the comparison of each compound treatment condition with DMSO. p-values were calculated using the Mann Whitney U test.

As the imaging assay is only a proxy for mycobacterial growth, selected compounds representing each of the major five classes were retested using the gold-standard for mycobacterial growth, colony-forming units (CFU). Compounds repurchased from commercial sources were tested at multiple concentrations to demonstrate the dose-dependence of growth-inhibition. J774 murine macrophages were infected with *M. tuberculosis* strain H37Rv, treated with three concentrations of each compound, and allowed to grow for three days. Prior to harvesting cells, we verified microscopically that there was no significant macrophage toxicity. Cells were then washed with PBS, lysed, and plated for CFU. Relative to dimethylsulfoxide (DMSO) controls, fluoxetine (a selective serotonin reuptake inhibitor; membrane transport protein), farnesyl thiotriazole (FTT) (a protein kinase C activator; kinase modulator), AKTi1/2 (an inhibitor of PKA/PKB; kinase modulator), quinidine (a sodium channel inhibitor; ion channel inhibitor), and ritanserin (an antagonist at the serotonin 2A receptor; GPCR modulator) all demonstrated significant dose-dependent activity against intracellular mycobacteria. At the maximum concentrations used, inhibition ranged from 50% for FTT to 75% for fluoxetine (Figure 2A). While this is less than would be expected from a traditional antibiotic with direct bactericidal activity, it is consistent with the magnitude potentially expected when host regulators of infection are targeted [10], [11]. A subset of compounds were additionally retested for activity in 5 point dose-response using our imaging assay (Figures S3, S4, S5, S6, S7, S8); observed inhibition was similar to that seen using CFU.

Because immortalized cell lines may contain mutations that alter signaling pathways and gene expression, we next verified that these same compounds have similar activity in a primary macrophage model. Primary mouse bone marrow-derived macrophages (BMDM) were infected with H37Rv and treated with the same panel of bioactive compounds. Because mycobacteria grow more slowly in BMDM than in cell lines, infection was allowed to progress for 5 days. After light microscopy was used to confirm the absence of macrophage death, cells were washed and lysed, and then released bacteria were plated for CFU. All tested compounds had significant activity against *M. tuberculosis* in BMDM (Figure 2B).

To confirm that the bioactive compounds were acting primarily on the host cells and were not directly toxic to the bacilli at the concentrations used in the macrophage infection model, compounds were tested for growth inhibitory activity against *M. tuberculosis* growing in axenic culture. At concentrations higher than the highest concentration used against infected macrophages, none of the inhibitors had significant activity against *M. tuberculosis* growing in axenic culture at any point during a 14-day time course (Figure S9).

### Analysis of immune regulatory pathways in infected macrophages treated with host-targeted inhibitors

**Figure 3:**
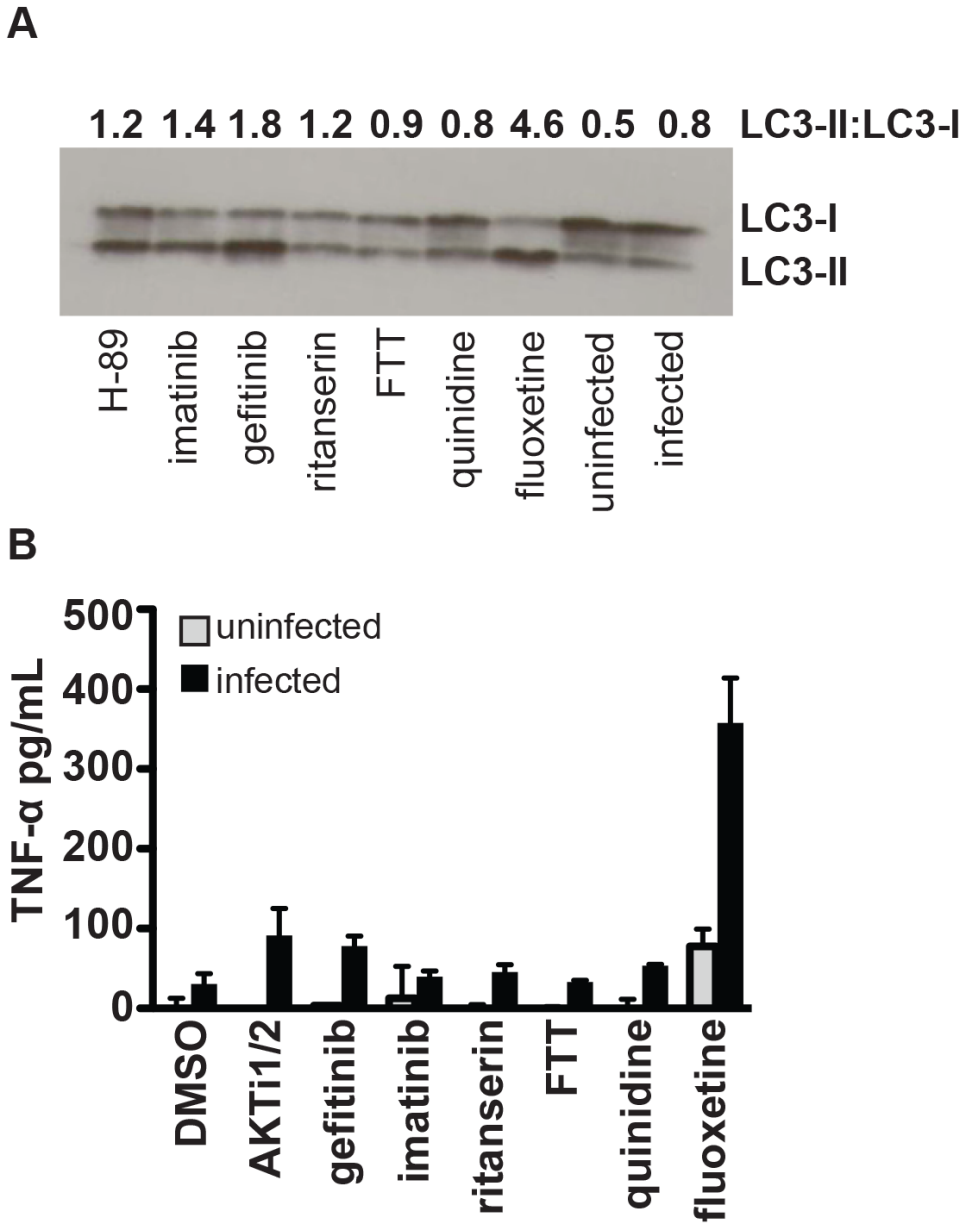
Specific compounds enhance autophagy or inflammatory cytokine release. (A) J774 murine macrophages were infected with H37Rv, then treated with the indicated compounds. Cells were harvested for Western-blot analysis of LC3 conversion from LC3-I to LC3-II as an indicator of autophagy. Data represents one of three independent experiments. Densitometry for LC3-II to LC3-I ratio for the shown Western blot was performed using ImageJ image analysis software. (B) Cells were infected with H37Rv, then treated with compound after a 4 hour phagocytosis. Supernatants were collected at 24 hours after infection and TNF-α concentration was determined by ELISA. Points represent average for 2 wells +/− standard deviation. Data represents one of two independent experiments.

Host targeted inhibitors that restrict the ability of *M. tuberculosis* to proliferate in a macrophage could function in various ways, such as by activating or enhancing a functional immune response like autophagy, or by inhibiting a signaling pathway activated by *M. tuberculosis* as a virulence mechanism. As a recent genome-wide RNAi screen that sought to identify host factors that regulate the ability of *M. tuberculosis* to replicate in macrophages predominantly identified negative regulators of autophagy [10], we tested whether our small molecule inhibitors might function by activating autophagy. LC3 is a ubiquitin-like protein that localizes to autophagolysosomes and is used as a specific molecular marker of autophagy. Upon induction of autophagy, LC3-I is converted to LC3-II by lipidation by a ubiquitin-like system; this conversion can be used as a readout of autophagy. Using the LC3 conversion assay, we determined that relative to uninfected macrophages or infected macrophages treated with DMSO control, both the EGFR inhibitor gefitinib and the serotonin transport inhibitor fluoxetine significantly enhanced autophagy (Figure 3A). Although the degree of LC3 conversion varied from experiment to experiment, these inhibitors consistently increased the LC3-I to LC3-II ratio. Other small molecules, including AKTi1/2, imatinib, quinidine, and ritanserin, did not consistently or significantly enhance LC3 conversion. These results suggest that gefitinib and fluoxetine restrict intracellular mycobacterial growth at least in part by enhancing host autophagy pathways. As the two molecules are not structurally related and act within distinct signaling pathways, there may be multiple routes through which autophagy can be activated to restrict the growth of *M. tuberculosis*.

### Inflammatory responses of macrophages are enhanced by treatment with fluoxetine

The capacity to mount an inflammatory cytokine response to infection is critical to the host capacity to control tuberculosis. Patients with latent tuberculosis treated with TNF-α inhibitors or soluble receptors are at significantly increased risk of developing reactivation disease [22], [23]. Similarly, patients with mutations in the IFN-γ receptor are at increased risk of severe mycobacterial disease [24]. On a cellular level, infection of macrophages with bacterial pathogens results in the induction of inflammatory responses that can ultimately lead to control of infection. *M. tuberculosis* infection of unactivated macrophages results in an induction of cytokines including TNF-α and the production of reactive oxygen and nitrogen species; however, in the absence of IFN-γ activation, the response is insufficient to control replication. Host targeted inhibitors could limit intracellular proliferation by upregulating TNF-α, a cytokine which has been shown to inhibit mycobacterial growth in macrophages through both nitric oxide dependent and independent mechanisms [25]. To determine whether treatment of infected macrophages with our inhibitors results in upregulation of TNF-α, macrophages were infected with *M. tuberculosis* and TNF-α levels were measured in the supernatants 24 h after infection. We found that treatment of infected macrophages with fluoxetine led to a significant increase in the amount of TNF-α produced (Figure 3B). This result is consistent with previous reports that serotonergic drugs including fluoxetine increase the *in vivo* levels of inflammatory cytokines, including TNF-α, both in human subjects and in animal models [26]. Thus, the effects that we observe in cell culture may be generalizable to a whole organism. Induction of autophagy has been described among the effects of TNF-α on macrophages [27]. As fluoxetine both induces TNF-α and autophagy, fluoxetine may induce autophagy by increasing levels of TNF-α. These results suggest that host-targeted therapies have the potential to inhibit *M. tuberculosis* proliferation by enhancing host protective inflammatory responses, at least at the level of the individual macrophage. Given the widespread clinical use and safety profile of serotonergic drugs such as the SSRIs, further investigation on whether their administration could impact tuberculosis treatment would be interesting.

### Targeting host kinases to inhibit intracellular mycobacterial growth

Kinases are important signaling molecules that regulate many aspects of mammalian cell biology including functions important for cellular response to infection, such as innate immune pathways, apoptosis, and autophagy. Kinase signaling has previously been demonstrated to be important for supporting *M. tuberculosis* replication in macrophages [11]. We identified 15 compounds that are annotated as kinase inhibitors in our screen (Table S1). Several of the targets of these kinase inhibitors have been previously implicated in the pathogenesis of *M. tuberculosis* or related pathogenic mycobacterial species, validating the capacity of the screen to identify biologically meaningful results [10], [12], [28]. H-89, an ATP-competitive inhibitor of kinases including both AKT and PKA, has been shown to inhibit proliferation of *M. tuberculosis* in mouse bone marrow derived macrophages [10], [12]. Imatinib, an inhibitor of Ableson family kinases (ABL) was also recently demonstrated to decrease the ability of *M. tuberculosis* to replicate in both macrophages and mice [28], [29]. However, the specific role that these kinases play in supporting replication of *M. tuberculosis* in host cells is unclear.

**Figure 4:**
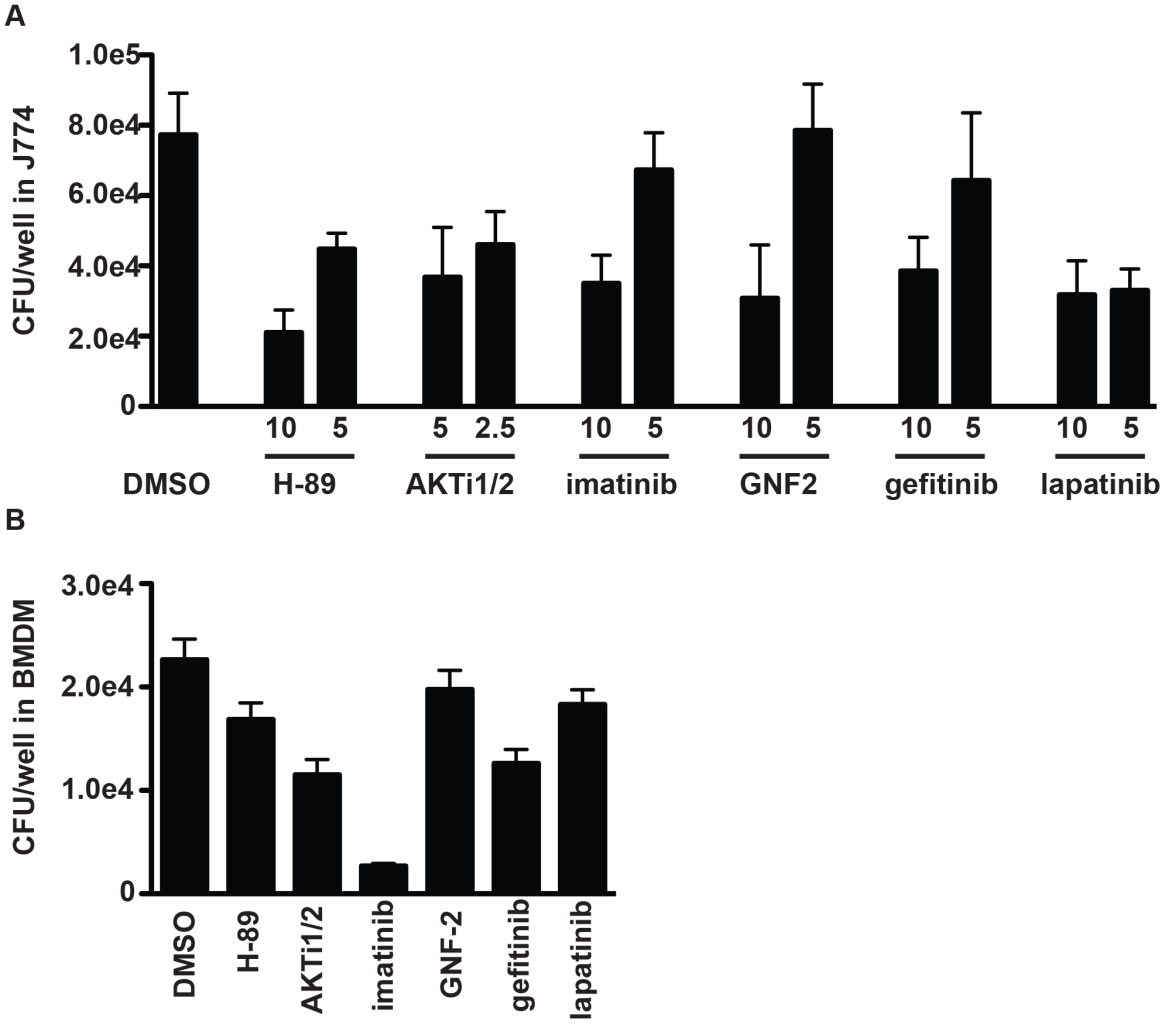
Inhibitors of protein kinases impair mycobacterial growth in macrophages. (A) J774 cells or (B) BMDM were infected with *M. tuberculosis* strain H37Rv at an MOI of 1∶1, and treated with each kinase inhibitor at the indicated concentrations (µM) after a 4 h phagocytosis period. At day 3 (J774) or day 5 (BMDM) after infection, cells were washed, lysed, and plated for CFU. Each column represents the mean and standard deviation of four biological replicates and each graph represents one of three independent experiments. For (A) all p<0.001 with the exception of GNF-2 at 2.5 µM, imatinib at 5 µM and gefitinib at 5 µM. For (B) inhibitors were used at the following concentrations: AKTi1/2 5 µM (p<0.03), H-89 5 µM (p<0.03), GNF-2 10 uM (not significant), imatinib 5 µM (p<0.03), gefitinib 5 µM (p<0.03), lapatinib 5 µM (p = <0.06). All p values were calculated using the Mann Whitney U test.

Understanding the role that host kinase signaling has upon *M. tuberculosis* macrophage growth is important for understanding the basic biology of infection, but might also have significant implications for the development of host-targeted therapeutics. Several of the kinase inhibitors identified in our screen are compounds currently used clinically for non-infectious disease related indications, raising the possibility of repurposing of such drugs for the treatment of tuberculosis. To confirm the efficacy of kinase inhibitors, we focused on three kinases for which inhibitors are currently in clinical use or development for cancer treatment: AKT, ABL and Epidermal growth factor receptor (EGFR). For each of these kinases we identified and tested at least two structurally unrelated compounds annotated as specific inhibitors of the individual enzymes. Although in practice a target responsible for any given phenotype is not necessarily the annotated target, the correlation between structurally unrelated compounds annotated to have the same target increased our confidence in compound specificity. Treatment of *M. tuberculosis*-infected macrophages with inhibitors targeting AKT (AKTi1/2, H-89), Abl (imatinib, GNF2), or EGFR (gefitinib, lapatinib) lead to a >2 fold decrease in bacterial load at the highest tested concentration of each inhibitor in both J774 macrophages (Figure 4A) and mouse bone marrow macrophages (Figure 4B).

### AKT/PKB and *M. tuberculosis* infection of macrophages

**Figure 5:**
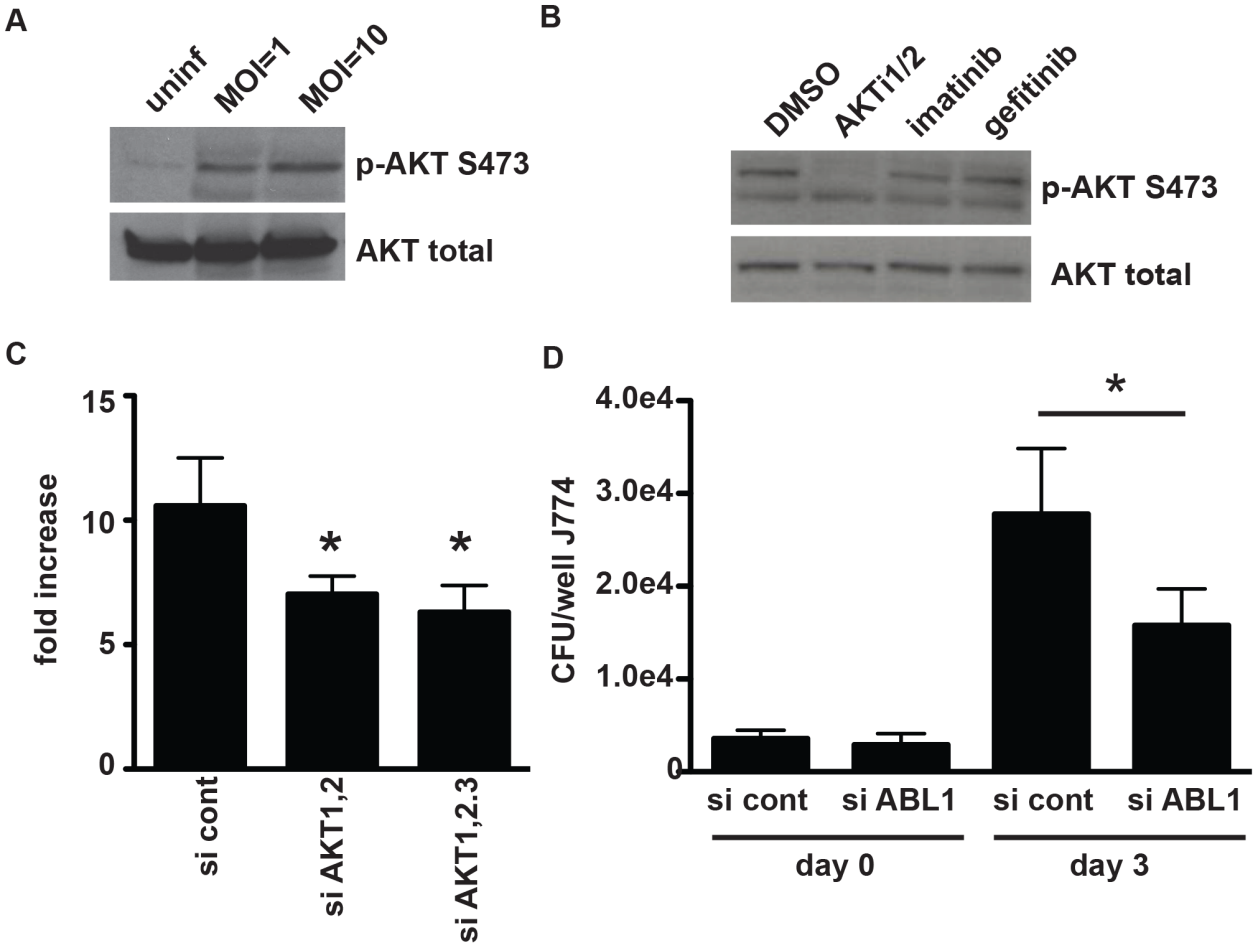
AKT and ABL are important for mycobacterial infection of macrophages. (A) J774 cells were infected with H37Rv at an MOI of 1 or an MOI of 10. Cells were harvested two hours after infection, and lysates were probed for AKT activation using an antibody to phospho-serine at position 473. (B) J774 cells were infected with H37Rv at an MOI of 1 for 4 hours, then washed and treated with compound. Cells were harvested at 3 hours after treatment and lysates were probed for AKT activation using an antibody to phospho-serine at position 473. (C) *akt1* and *akt2* or *akt1*, *akt2*, and *akt3* were silenced with siRNA in J774 cells. Cells were then infected with H37Rv at an MOI and 1, and infection was allowed to progress for 3 days. Cells were then lysed and plated for CFU. (D) *abl1* was silenced in J774 macrophages using siRNA. Cells were then infected with H37Rv. After 4 hours of phagocytosis, wells were lysed and plated for CFU to determine uptake (day 0). Infection was allowed to progress in the remaining wells; day 3 after infection, cells were lysed and plated for CFU. Each experiment was repeated a minimum of three times, and a representative experiment is shown. Error bars are standard deviation, *p = 0.0195 by Mann Whitney U for (C) and (D).

The serine/threonine kinase AKT/PKB has been previously implicated in the ability of *M. tuberculosis* and other bacterial pathogens to replicate in host cells [10], [12]. Treatment of infected cells with the AKT1 inhibitor H-89 was shown to limit proliferation of *M. tuberculosis* in infected mouse macrophages [12] and knocking down AKT1 and AKT2 in human THP-1 cells also led to decreased replication of intracellular *M. tuberculosis* [10]. In our screen, in addition to previously identified ATP competitive inhibitor H-89, which preferentially targets PKA in addition to AKT, we found that a more specific allosteric inhibitor of AKT, AKTi1/2 [30], [31] also restricts intracellular *M. tuberculosis* replication. To confirm that AKT plays a role in *M. tuberculosis* infection of macrophages, we first tested to see whether AKT was activated by infection with virulent *M. tuberculosis*. AKT activation requires phosphorylation at two residues, Thr-308 and Ser-473 [32]; Western blot analysis for this phosphorylation is a standard assay for activation. Using Western blot analysis for phosphorylation of Ser-473, we observed rapid activation of AKT upon infection of J774 macrophages with *M. tuberculosis* (Figure 5A). Importantly, the activation of AKT was completely abrogated by treatment with the allosteric inhibitor AKTi1/2 that is known to block the phosphorylation and activation of AKT1 and AKT2 (Figure 5B). To genetically confirm the role of various AKT isoforms in macrophage infection, we knocked down the expression of AKT1, AKT2, and AKT3 in J774 macrophages (Figure S10). Similar to previously reported results [10], we found that the maximum effect was observed with simultaneous knockdown of AKT1 and AKT2. We also found that knocking down AKT3 provided little additional benefit (Figure 5C). That silencing both AKT1 and AKT2 is required for maximal effect may account for the failure to identify AKT in the original RNAi screens that target only one isoform at a time, while a small molecule inhibitor inhibits both isoforms simultaneously.

### The role of ABL tyrosine kinase during infection with *M. tuberculosis*

Imatinib mesylate (Gleevec, STI-571) is a tyrosine kinase inhibitor that is currently used therapeutically for treating chronic myelogenous leukemia (CML). Imatinib inhibits the ABL family tyrosine kinases ABL1 and ABL2 and other related tyrosine kinases [33]. In fact, imatinib's activity against CML may require activity at more than one target [34]. Recently, imatinib was shown to diminish entry of *Mycobacterium marinum* in mouse fibroblasts and *M. tuberculosis* in mouse macrophages [28]. In addition, imatinib treatment decreased bacterial replication in *M. marinum* and *M. tuberculosis* infections of mice [28]. Identification of the ABL inhibitors GNF-2 and imatinib in our screen, which was designed to avoid inhibition of initial phagocytosis, supports the idea that ABL is important not simply for cell entry but also for later replication and survival of mycobacteria in macrophages. A subsequent study has shown that imatinib increases acidification of the lysosomal compartment of macrophages. This effect was required for the anti-mycobacterial activity of the compound [29].

As imatinib inhibits both ABL1 and ABL2, in addition to other tyrosine kinases, the relevant target during *M. tuberculosis* infection of macrophages is not clear. Further, in apparent contradiction, silencing of *abl1* using siRNA has been reported to increase *M. tuberculosis* replication in macrophages [11] suggesting that this family of kinases may play complicated roles during infection. We therefore sought to characterize the role of ABL1 in entry and survival of *M. tuberculosis* in macrophages. To specifically abrogate ABL1 function, we transfected macrophages with siRNA targeting *abl1*. Although we observed significant silencing of ABL1 expression (Figure S10), we observed no difference in *M. tuberculosis* entry into cells treated with *abl1*-specific siRNA, demonstrating that ABL1 may not in fact play a role in *M. tuberculosis* entry (Figure 5D, Figure S10B). However, after three days of infection we observed a ∼1.9 fold decrease in bacterial replication in cells treated with siRNA targeting *abl1* compared to cells treated with a non-specific siRNA control, indicating that ABL1 likely plays a role in *M. tuberculosis* intracellular survival post entry into macrophages (Figure 5D). Importantly, knocking down expression of only ABL1 gave approximately the same magnitude effect observed in imatinib treated cells (∼2.1 fold), suggesting that the effects of imatinib are likely mediated primarily through ABL1 during *M. tuberculosis* infection of macrophages.

### The role of epidermal growth factor receptor during infection with *M. tuberculosis*

Although primarily studied in the context of cancer, EGFR has been linked to influenza uptake [35], to regulation of inflammation following rhinovirus infection [36], and to prevention of apoptosis in host cells in bacterially infected gastric epithelial cells [37]. EGFR has not previously been linked to mycobacterial infection. We identified gefitinib (Iressa, ZD-1839), an EGFR inhibitor used for treating non-small cell lung cancer [38], and two other EGFR inhibitors in our primary screen (Table S1). Subsequent testing in J774 macrophages and BMDM confirmed that gefitinib treatment controls *M. tuberculosis* intracellular growth (Figures 4A–4B); we confirmed that EGFR is in fact expressed in macrophages using reverse transcription and PCR-based detection of transcript (Figure S11).

**Figure 6:**
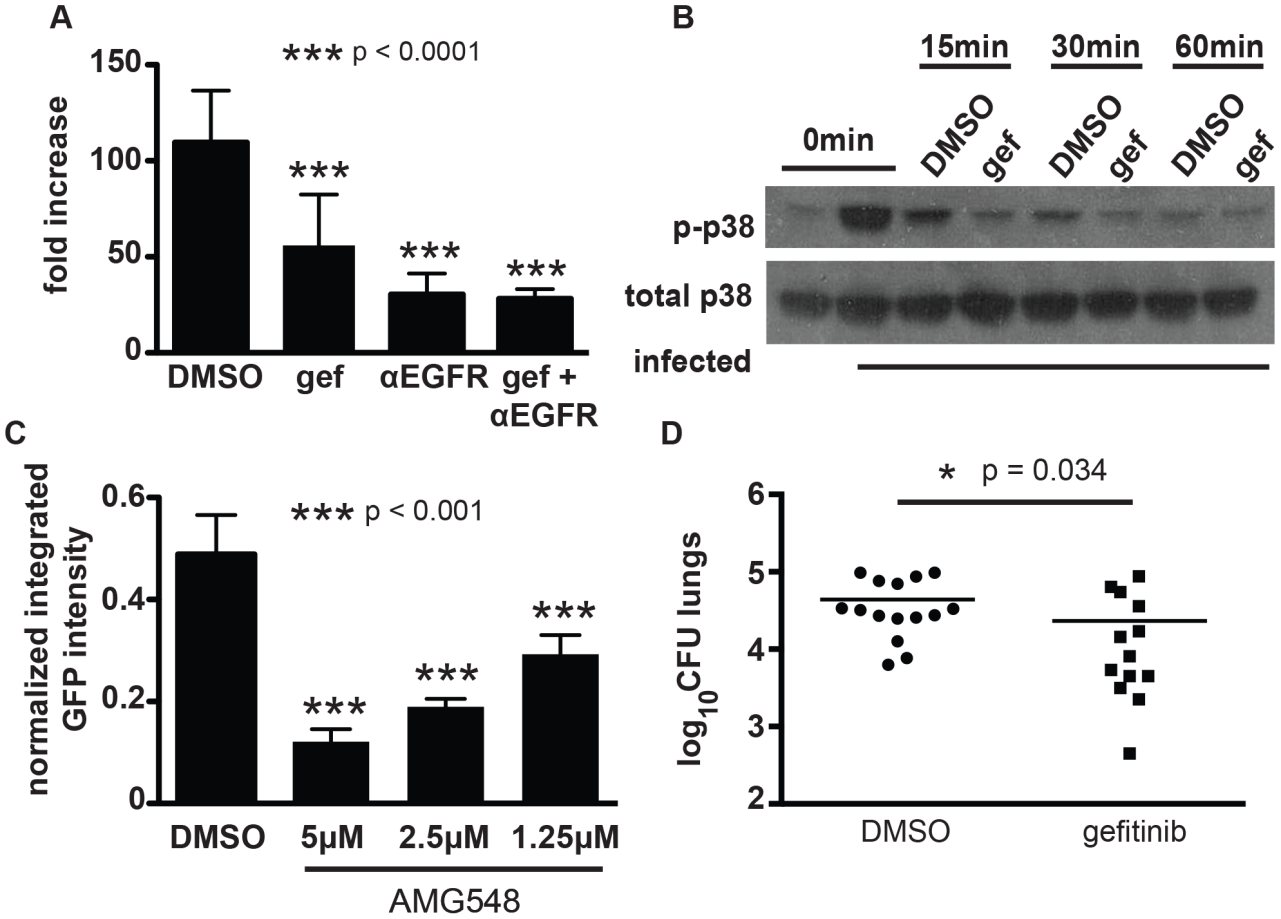
EGFR is important for mycobacterial infection of macrophages and in a mouse model of infection. (A) human primary PBMC derived macrophages were infected with a luciferase-expressing Erdman strain, then treated with EGFR-neutralizing antibodies (αEGFR) and/or gefitinib at 10 µM (gef) 4 h after the phagocytosis period. Antibodies and inhibitor was refreshed daily, and bacterial growth was determined by luminescence on day 5 after infection. The data is representative of two independent experiments. (B) J774 cells were infected with *M. tuberculosis* strain H37Rv. 4 hours after infection, cells were washed and gefitinib or DMSO was added. Cells were lysed at 15 minutes, 30 minutes, or 60 minutes after drug treatment and lysates were probed for phospho-p38 and total p38 by Western blot. The data is representative of three independent experiments. (C) J774 cells were infected with H37Rv-GFP. 4 hours after infection, cells were washed and DMSO or AMG548 was added at the indicated concentrations. Day 3 after infection, cells were washed and fixed with 4% paraformaldehyde. Plates were imaged and analyzed using our CellProfiler pipeline to determine integrated GFP intensity normalized to macrophage number. The data represent one of two independent experiments. (D) Mice were infected with *M. tuberculosis* strain Erdman via the aerosol route with ∼200 CFU and infection was allowed to progress for 7 days. Beginning day 8 after infection, mice were treated with DMSO or gefitinib 100 mg/kg by intraperitoneal injection daily for six days. Day 14 after infection, mice were sacrificed and lungs were plated for CFU. Data combined from three independent experiments is shown. ***p<0.001, *p = 0.034 by non-parametric Mann-Whitney U test. Error bars are standard deviation.

To confirm that the small molecules function by specifically inhibiting EGFR signaling, we sought to perturb EGFR signaling in an alternative manner. As EGFR is a well-validated target for anticancer therapy, several monoclonal antibodies for blocking human EGFR have been developed. In particular, treatment of cells with combinations of non-competitive antibodies against EGFR results in synergistic receptor downregulation via recycling inhibition in a manner that does not result in activation of EGFR signaling [39]. We first confirmed that gefitinib effectively blocks *M. tuberculosis* replication in primary human monocyte derived macrophages using a total of four donors (data not shown). Next, we treated infected human macrophages with two EGFR-neutralizing antibodies [39]. Treatment with neutralizing antibodies inhibited intracellular *M. tuberculosis* growth to the same degree as treatment with gefitinib. (Figure 6A), confirming the role of EGFR in *M. tuberculosis* intracellular survival and replication in primary human macrophages.

### Gefitinib inhibits phosphorylation of the downstream signaling molecule p38 after infection

There are several signaling pathways that can be triggered downstream of EGFR activation, including activation of AKT, ERK, JNK, and MAPK p38. To determine whether the effect of EGFR inhibition was mediated by any of these signaling pathways, we first sought to determine whether they were activated by *M. tuberculosis* infection of macrophages. Both AKT and p38 were phosphorylated upon infection (Figures 5A and 6B), consistent with activation. ERK and JNK did not appear to have increased phosphorylation upon infection (data not shown). Surprisingly, AKT phosphorylation was not inhibited by gefitinib treatment (Figure 5B), suggesting that the effect of inhibiting EGFR during *M. tuberculosis* infection is independent of AKT signaling. To determine whether p38 signaling is impacted by gefitinib, we measured phosphorylation of p38 and found that it was consistently inhibited in the presence of gefitinib treatment (Figure 6B). While p38 phosphorylation decreased over time after infection even in the absence of compound, gefitinib-treated cells had significantly less p38 phosphorylation than DMSO-treated cells at multiple timepoints. To determine whether inhibition of p38 phosphorylation could be the mechanism by which EGFR inhibition of gefitinib treatment restricts *M. tuberculosis* growth in macrophages, we tested whether selective p38α inhibitor AMG548 [40] would also restrict intracellular mycobacterial growth. We found that inhibition of p38α by AMG548 does in fact restrict mycobacterial growth in a dose-dependent fashion (Figure 6C). MAPK p38 has been demonstrated to regulate autophagy via p38IP and mATG9, with signaling through p38 associated with inhibition of autophagy and depletion of p38 with activation of autophagy [41]. Consistent with our finding that gefitinib induces autophagy (Figure 3A), this report that p38 inhibition can induce autophagy [41], and our implication of p38 downstream of EGFR activation after *M. tuberculosis* macrophage infection, we suggest that inhibition of p38 activity downstream of gefitinib-mediated EGFR inhibition may trigger an increase in autophagy within the cell, resulting in enhanced clearance of *M. tuberculosis*.

### Gefitinib is effective for treating *M. tuberculosis* in vivo

As there are few studies implicating EGFR in bacterial pathogenesis, we sought to determine whether EGFR signaling is relevant to *M. tuberculosis* replication *in vivo*. Given the problem of drug-resistant tuberculosis and the long courses of therapy currently required for treatment, there is growing interest in host-directed therapies as adjunctive therapy, with the potential to shorten therapy and increase the efficacy of current antitubercular antibiotics [11], [17], [28], [42]. To determine whether gefitinib has efficacy against *M. tuberculosis in vivo*, we examined the efficacy of this inhibitor during early acute infection with *M. tuberculosis* in the mouse model. We chose to test during early infection prior to the recruitment of IFN-γ producing T cells to the lungs, as this is the time period that is best modeled by *in vitro* infection of unactivated macrophages. BALB/c mice were infected with *M. tuberculosis* via aerosol inoculation. Infection was allowed to progress for 7 days prior to initiation of treatment. Mice were subsequently treated with gefitinib at a dose of 100 mg/kg every day for six days. On day 14 after infection, 7 days after initiation of gefitinib treatment, mice were sacrificed, and lungs were homogenized and plated for CFU. The experiment was performed three times and the combined results are shown. Relative to DMSO-treated control mice, mice treated with gefitinib had statistically significantly less bacterial burden (Figure 6D). These results support the *in vivo* relevance of EGFR in infection and the idea that host-targeted therapies, and kinase inhibitors specifically, may be useful as adjunctive treatment for tuberculosis. Further work will clearly be required to demonstrate efficacy in additional, more chronic models of *M. tuberculosis* as well as to determine any potential benefit when used in combination with traditional anti-tuberculosis chemotherapeutic agents.

## Discussion

We have developed a high-content, high-throughput imaging assay to identify small molecules that inhibit the growth of *M. tuberculosis* within macrophages. We applied our assay to screen a library of known bioactive molecules, and from that screen identified several compound classes with diverse annotated targets that result in reproducible, dose-dependent intracellular *M. tuberculosis* growth restriction. Out of the compounds of interest in our five primary categories, several hits validated previously described targets [12], [28], while others pointed to the involvement of potentially novel factors important for infection, including EGFR, the serotonin transporter targeted by SSRIs, serotonin and dopamine receptors, and sodium channels. An important caveat is that an annotated mechanism of action may not be relevant to the actual activity of a compound in a given assay; observed activity may be due to some other “off-target” effect. Thus, we have used complementary genetic approaches to validate a few exemplary small molecules and implicate their annotated targets in our identified phenotype of *M. tuberculosis* growth restriction within macrophages.

Three previous chemical screens had identified compounds that restrict *M. tuberculosis* growth within the context of macrophages. The first, by Christophe et al., focused on screening compounds that are predominantly without known activities [15]. Moreover, the compounds selected for follow-up target essential bacterial processes rather than host proteins relevant to the host-pathogen interaction. A similar screen by the same group also focused on compounds without known activities that targeted essential bacterial processes [16]. A third chemical screen was, like our screen, skewed toward bioactive compounds that target host proteins [17]. Of our 133 identified active compounds, 21 were screened by Sundamarthy *et al.*, and of those compounds 13 were identified as active by their criteria. While distinct libraries screened explain some of the differences between the lists of hits, more subtle differences contribute as well. Of the 8 compounds identified in our screen that were screened but not identified as hits in the screen by Sundamarthy *et al.*, several score close to their threshold for consideration as a positive result. The discrepancies for those compounds likely reflect differences in the precise cutoff for determining hits and the sensitivity of the respective assays. However, some clear hits from our screen scored poorly in their assay, possibly reflecting biological differences between the assays. While our assay used *M. tuberculosis* and was designed to identify a visual output that approximated differences in mycobacterial growth as optimized using typical antimycobacterial agents, the assay used by Sundamarthy *et al.* used Bacille Calmette Guerin and had a complex visual output based on phenotypic changes including host cell morphologic changes, phenotypes felt to represent toxicity to the host cell, bacterial size, bacterial number, and bacterial intensity. In fact, they report that traditional antimycobacterial agents did not perform well in their screen, suggesting that it was not designed primarily to identify molecules that affect bacterial load when compared to the gold standard of CFU. Their phenotype likely represented growth restriction mediated by the particular host processes captured by their visual output, such as enhanced autophagy, rather than overall growth restriction. Overlapping hits between the screens may all have a similar mechanism of growth restriction, while compounds that were hits in our screen but not in the screen by Sundamarthy *et al.* may have distinct mechanisms of action.

We identified several ion channel inhibitors in our screen, including niguldipine and verapamil, which block calcium channels, and flecainide and quinidine, which block sodium channels. A role for voltage-gated calcium channels in mycobacterial infection has previously been suggested [43]. In addition to altering calcium signaling, at least one calcium channel blocker, verapamil, has been shown to inhibit a bacterial efflux pump that is important in the context of intracellular infection [44]. Multiple calcium channel blockers, including verapamil, are used extensively in the clinic for cardiac applications. Our work adds to the growing body of work that suggests a role for calcium channel blockers as adjunctive therapy. In fact, in a recent paper, Gupta *et al.* tested the addition of verapamil to the standard background regimen of isoniazid, rifampin, and pyrazinamide to treat tuberculosis in a murine model, and found that addition of verapamil both reduced CFU during the active phase of infection and reduced rates of relapse [45]. Whether these reductions are the result of the effect of verapamil on the host or the bacterium is unclear. Nevertheless, this work validates the possibility that compounds identified in this screen may ultimately be useful as adjunctive agents for the treatment of tuberculosis. A potential role for sodium channels has not previously been described and merits further investigation.

Our second class of targets, GPCRs, has only recently been suggested to have a role in mycobacterial infection. While recent work implicated a single GPCR, the D-3-hydroxybutyrate receptor GPR109A [46], our work identified primarily inhibitors of central nervous system-associated GPCRs, such as serotonin receptors and dopamine receptors. Although their function in macrophages is not well-understood, several central-nervous system associated GPCRs have been described to be expressed in macrophages [47], [48], [49]. While some of these receptors have previously been shown to have a role in modulating inflammatory cytokines in response to specific stimulation [50], [51], none have explicitly been shown to be involved in infection or the host-pathogen interface. Our results raise the intriguing possibility that these receptors play roles in the host immune response that are quite distinct from their roles in the central nervous system.

The membrane transport proteins identified in this screen, including the serotonin transporter targeted by SSRIs, a dopamine transporter, and an acetylcholine transporter, have similarly not been shown to be involved in infection previously. Fluoxetine has been noted to increase systemic TNF-α [26], consistent with our results. The role of TNF-α during infection with *M. tuberculosis* is complex. Low TNF-α levels are clearly detrimental for the host and lead to impaired control of bacterial replication. However, overproduction of TNF-α can have host detrimental effects resulting from excessive tissue damage, induction of macrophage necrosis, and potentially from signaling bacteria to enter a nonreplicating antibiotic tolerant state [52], [53], [54]. Whether the levels of TNF-α produced during infection of humans with *M. tuberculosis* are optimal for bacterial replication or for host protection is not clear, and this likely varies with the genotype of both the infecting strain and the infected individual. Tobin et al. demonstrated in a zebrafish model of *M. marinum* infection that particular host genotypes of LTA4H resulted in either high or low levels of TNF-α production in response to infection, either of which was detrimental for outcomes. They went on to demonstrate that promoter polymorphisms for LTA4H in humans similarly resulted in high or low levels of TNF-α production. In an elegant translational component of their study, they demonstrated that among patients with tuberculosis meningitis, only those with the high- TNF-α producing genotype benefited from the standard addition of corticosteroids to their therapeutic regimen [52]. Conversely, it is likely that individuals that produce low levels of TNF-α upon infection with *M. tuberculosis* may benefit from adjunctive therapy that enhances production. Given the safety profiles of SSRIs, their widespread use, and their ability to modulate TNF-α levels and induce autophagy during infection, they are attractive candidates for further exploring the possibility of tailoring TNF-α levels to optimize host response in the infected individual.

The bulk of the compounds in our anti-inflammatory category were non-steroidal anti-inflammatories (NSAIDs). Their identification is somewhat surprising as their inhibition of cyclooxygenase should block the production of a metabolite, prostaglandin E2, previously shown to be protective for infected macrophages and to reduce the bacterial burden in infected cells [5]. In fact, one would expect an increase in metabolites generated by competing pathways, including lipoxygenase-mediated production of lipoxins such as lipoxin A4, which has been shown to impair the host response to infection [5]. Thus, an NSAID-induced shift in metabolic balance away from prostaglandin E2 toward lipoxins might be expected to worsen the outcome of infection. In contrast, the identification in our screen of multiple distinct NSAIDs that restrict *M. tuberculosis* growth in macrophages suggests that the role of eicosanoid pathways in mycobacterial infection is potentially more complex than we understand thus far. Given how inexpensive, well-studied, and generally well-tolerated NSAIDs are, they certainly merit further study in animal models of infection. Corticosteroids were also represented in our anti-inflammatory category. Anti-inflammatory corticosteroids have been used as adjunctive therapy for tuberculosis for decades [55]. The use of corticosteroids for tuberculosis meningitis has been accepted into clinical practice; however, the potential benefit of adjunctive steroids for any other form of tuberculosis has been a matter of debate within the literature. A recent meta-analysis found that steroids significantly reduced mortality associated with tuberculosis infection of all organ groups [56]. Our finding that corticosteroids reduce bacterial burden in a cellular model of infection raises the question of whether steroids might impact infection at the level of infected macrophages that counteract the immunosuppressive effects on the whole organism level.

We identified multiple kinase inhibitors in our screen. The identification of AKT and ABL family inhibitors validated our screen, as inhibitors of both classes have been previously shown to disrupt mycobacterial proliferation in macrophages. While a previous siRNA screen had identified AKT indirectly through association with genes identified in their screen [10], AKT was not directly identified using siRNA. Our study demonstrates the inherent advantage of small molecules over RNAi in simultaneously targeting multiple redundant isoforms of a given protein. Imatinib, an inhibitor of ABL family kinases, had similarly been demonstrated to be important for mycobacterial infection of macrophages, predominantly by both inhibiting bacterial uptake and subsequent replication [28]. Using primarily a model with *M. marinum*, which has significant differences from *M. tuberculosis* in its intracellular lifestyle, Napier et al. found that the effects of imatinib were to restrict *M. marinum* uptake and limit its intracellular growth, thus implicating imatinib-sensitive tyrosine kinases as important for virulence. In contrast, a previous study targeting ABL1 with siRNA suggested that depletion of ABL1 results in increased proliferation of *M. tuberculosis* in macrophages [11]. Here we demonstrate that ABL1 is specifically required for proliferation of *M. tuberculosis* in macrophages. Our study does not show reduced uptake of *M. tuberculosis* in cells treated with imatinib or with reduced ABL1 expression. Instead, reduction of ABL1 expression or inhibition of ABL function seems to specifically restrict intracellular growth at a later stage.

Finally, we identified EGFR/p38 MAPK signaling pathway as a novel regulatory pathway during mycobacterial infection that functions to suppress effective antimicrobial responses. Inhibition of EGFR appears to restrict growth of intracellular mycobacteria through induction of autophagy in an AKT-independent mechanism, potentially through downstream inhibition of p38 MAPK. Previous studies have demonstrated a role for p38 MAPK as a negative regulator of both basal and starvation-induced autophagy via p38IP and mATG9 [41]. Our data suggests that EGFR signaling during *M. tuberculosis* infection activates a similar p38 dependent pathway that prevents clearance of the bacteria by autophagy. Using an EGFR inhibitor in a murine model of infection, we show the relevance of EGFR signaling *in vivo* and demonstrate that targeting the host with compounds already in clinical use for other applications holds potential for novel therapeutics for tuberculosis. This work thus demonstrates a possible therapeutic strategy of targeting host factors that modulate intracellular *M. tuberculosis* infection and replication. Repurposing of agents already in clinical use for other indications could expedite the testing of this strategy for tuberculosis.

The general idea of modulating the host response to improve the outcome of tuberculosis treatment has circulated for some time. This concept however, is enormously complex as any benefit to the patient must integrate the impact of host-targeted intervention across all cell and tissue types and all systemic responses, and throughout the whole course of infection. Thus, benefits on a cellular, macrophage level may be counterbalanced by detrimental systemic effects involving numerous cell types and cytokine responses, or vice versa. Further, interventions with benefits at one point in infection, for example early in an inflammatory process, may not necessary be beneficial late in infection. Nevertheless, treatments that limit inflammation have been used both clinically and in experimental settings as adjunctive therapy for conventional antibiotics. Treatment of human patients with corticosteroids results in a modest decrease in mortality and is helpful in some forms of extrapulmonary tuberculosis including meningitis and pleural disease [56], possibly by limiting host inflammatory related tissue damage and/or by allowing *M. tuberculosis* to transition into a state of active replication in which it is sensitive to antibiotics [57]. Similarly, while anti-TNF-α agents lead to reactivation of mycobacterial disease, at the same time, blocking TNF-α has been suggested to favor a host microenvironment that favors bacterial clearance, particularly in the face of tubercular chemotherapy. Specifically, agents that either directly block TNF-α or inhibit signaling mechanisms that indirectly result in TNF-α production have been used to enhance the responsiveness of bacteria to conventional antibiotics. The TNF-α blocking agent etanercept increased the efficacy of conventional antibiotics during the chronic phase of infection, when the bulk of the bacterial are thought to be replicating slowly [58]. FDA-approved phosphodiesterase (PDE) inhibitors, which alter intracellular levels of cAMP resulting in reduced TNF-α secretion, likewise have been shown to reduce bacterial burden in rabbit and mouse models of infection when combined with current antimycobacterial antibiotics [42], [59]. Of note, an inhibitor of PDE-4 enhanced the effect of isoniazid on clearance of bacteria, but did not have an effect alone [42]. Inhibitors of PDE-3 and PDE-5 were not tested in the absence of standard tuberculosis therapy, so whether they would have an effect alone is unknown [59].

In contrast to work studying therapies that potentially manipulate immunity at a systemic level, a growing body of literature including this current work supports the idea that targeting specific host-pathways to enhance molecular mechanisms for bacterial clearance on the cellular level may be an effective adjunctive strategy for treatment [60]. Based on known host-pathogen biology or the RNAi screens described above, a variety of host targets have been identified and tested in animal models. Targeting lipid metabolism, a liver X receptor inhibitor reduced bacterial burden in the lungs of mice infected with *M. tuberculosis* [61]. As noted above, imatinib, an ABL inhibitor, has been shown to reduce bacterial burden in a murine model of infection [28]. Additionally, a TGF-β receptor inhibitor [11] and a GPR109A inhibitor [46], selected for study based on findings from the RNAi screen of *M. tuberculosis* infected THP-1 cells, were both shown to be similarly effective in reducing the burden of disease in mice. As the models for infection and treatment differ from study to study, it is difficult to directly compare efficacy between studies. Unlike the PDE inhibitors however, the liver X receptor inhibitor, ABL inhibitor, TGF-beta inhibitor, GPR109A inhibitor, and now an EGFR inhibitor all have efficacy even in absence of traditional tuberculosis therapy. These results suggest that enhancing host mechanisms of intracellular killing is a viable option for novel TB therapies, while reiterating the need to move quickly from *in vitro* cellular models to infected animals to determine the effect of an intervention on the complex, intact immune response of a whole organism. Additionally, efforts to determine the timing during an infectious process or the host genetic background where host intervention might be most beneficial will be important to such approaches.

As the problems of MDR- and XDR-TB grow globally, identifying new therapeutic approaches will be critical for decreasing morbidity and mortality, and potentially disrupting the transmission of these highly resistant strains. Traditional drug development pipelines for anti-tuberculosis antibiotics have proven slow to move from lead compounds to clinically deployed medications. As several host-acting compounds that are already in clinical use with well-understood pharmacology and side effects have been shown to be effective in animal models of disease, such compounds could be rapidly tested in expanded animal models of infection and perhaps even moved to humans directly, given the limitations of existing animal models, to clarify the role of host-targeted therapies on treatment efficacy and duration. Alternatively, for some host-modulating compounds that are commonly used within patient populations (*i.e,* calcium channel blockers, NSAIDs, SSRIs), retrospective data may exist to provide initial support for any potential benefit of these host-targeted inhibitors. While some host-targeted therapies, including some kinase inhibitors, would currently be relatively expensive to administer, they are within the cost range of other medications being studied for use against for highly drug-resistant tuberculosis, including linezolid, and with time, their costs will drop as they become available off patent [62]. Others, such as some SSRIs or calcium-channel blockers, are currently inexpensive and would be amenable to inclusion in treatment regimens. Given the current state of tuberculosis, with rising incidence of MDR, XDR, and even TDR-TB, novel strategies are required that move beyond the conventional paradigm of an antibiotic that kills the bacterium in axenic culture. Targeting the host is one such strategy that can be tested as a feasible path forward exists, facilitated by the repurposing of current drugs.

## Materials and Methods

### Ethics statement

Animal work was approved by Massachusetts General Hospital IACUC (protocol number 2009N000203) or the Harvard Medical School HMA Standing Committee on Animals (protocol number 03000). All protocols conform to the USDA Animal Welfare Act, institutional policies on the care and humane treatment of animals, the “ILAR Guide for the Care and Use of Laboratory Animals,” and other applicable laws and regulations.

### Imaging assay protocol

J774 macrophages were seeded into 96-well black clear-bottom plates. *M. tuberculosis* strain H37Rv constitutively expressing GFP [18] was grown to mid-log phase in axenic culture, washed in PBS, briefly opsonized in heat-inactivated horse serum, and used to infect cells at an MOI of 1∶1. Infection was allowed to progress for four hours, then media was aspirated, cells were washed once with PBS, and media containing the screening compounds at an average concentration of 5 uM was added back to cells. Three days after infection, media was aspirated, cells were washed once with PBS, and fixed with 4% paraformaldehyde with Triton X-100 and DAPI.

### Image acquisition and analysis

Plates were imaged using an Image Xpress Micro high-throughput microscope (Molecular Devices). Images were taken with a 4× objective at four sites per well. Images were then analyzed using CellProfiler open-source software [19]. The imaging-analysis pipeline is openly available (http://cellprofiler.org/published_pipelines.shtml) and included correction to homogenize illumination over each field, a filter to remove any large debris from the analysis, identification and quantitation of DAPI-stained nuclei, identification, quantitation, and pixel intensity calculation for GFP-expressing bacteria. The final output was calculated as (average GFP pixel intensity per bacterium across the field)×(number of bacteria identified in the field)/number of nuclei per field. The four images sites per well were averaged. To identify hits we used p-values obtained by bootstrap Monte Carlo and composite z-scores (see Methods S1).

### J774 infections for CFU

J774 cells were seeded into 24 well plates. *M. tuberculosis* strain H37Rv was prepared as described above, then used to infect cells at an MOI of 1∶1. Phagocytosis was allowed to progress for 4 hours; cells were then washed once with PBS and fresh media containing compound was added back. Day 3 after infection, cells were washed once with PBS, lysed in 0.5% Triton X-100, and plated on 7H10 plates in serial dilutions.

### BMDM isolation and infections

Bone marrow was obtained from C57BL/6 mice. In brief, adult male mice were euthanized and femurs and tibias were harvested. Bone marrow was flushed from the cells, resuspended in DMEM, and plated non-tissue culture treated dishes in DMEM media containing 2 mM L-glutamine, 20% fetal bovine serum, and 25 ng/ml recombinant mouse M-CSF. Cells were harvested day 6 after bone marrow isolation and either plated for subsequent infection or frozen. For infections, cells were plated in 24 well plates and infected as above with H37Rv. Media was changed every two days. Day 5 after infection, cells were washed once with PBS, lysed with 0.5% Triton X-100, and plated for CFU.

### siRNA and blocking antibodies

For siRNA silencing experiments, J774 cells were plated in a 6-well dish. 20 pmol siRNA duplex was added in Optimem (Gibco) with 9 µl Lipofectamine RNAiMax (Invitrogen) at 24 h and again at 48 h after plating. The following day, cells were harvested, counted and re-plated for *M. tuberculosis* infections. 24 h after replating, the cells were infected with H37Rv as described above. To assess the efficiency of silencing, lysates were prepared at the same timepoint that the cells are infected with *M. tuberculosis*. For experiments blocking EGFR signaling, anti-EGFR antibody 225 (Millipore) and EGFR antibody Ab-5 (Thermo Scientific) were used at a final concentration of 20 nM. Media containing antibodies was replenished every 24 h.

### Macrophage assays

For monitoring LC3 conversion by Western blot analysis, 2×10^5^ J774 macrophages were plated per well in 12 well dishes and were infected with H37Rv at an MOI = 1. After 4 h the infected monolayer was washed once with PBS and media containing inhibitors was added. Three hours later the cells were washed, and protein lysates were prepared and run on a 15% SDS-PAGE gel. LC3-I and LC3-II were detected using an antibody from Cell Signaling Technologies. For TNF-α ELISAs, J774 or mouse BM macrophages were plated at 5×10^4^ cells per well in a 96 well plate and infected at an MOI = 1. Monolayers were washed with PBS after 4 h phagocytosis and media with inhibitors was added. Cell supernatants were collected 24 h later, and assayed for TNF-α using an ELISA kit (Invitrogen). For phospho-p38 and p38 westerns, J774 cells were infected with H37Rv at an MOI of 1. Phagocytosis was allowed to proceed for 4 hours. Cells were then washed with warmed media, and then treated with gefitinib or DMSO carrier. Cells were harvested at 0 minutes, 15 minutes, 30 minutes, and 60 minutes after drug treatment and probed for phospho-p38 or p38 using antibodies from Cell Signaling Technologies.

### Mouse infections

BALB/c mice were infected in a Madison aerosol chamber as previously described [63]. 5 mice were sacrificed day 1 after infection, and their lungs were homogenized and plated for CFU to determine the number of implanted bacteria. Infection in the remaining mice was allowed to progress for 7 days. Beginning day 8 after infection, mice were then given intraperitoneal injections of gefitinib at 100 mg/kg or DMSO carrier for 6 days. Day 14 after infection, 5 mice in each experimental group were sacrificed, and lungs were homogenized and plated for CFU.

## Supporting Information

Figure S1 **Positive controls for development of high-content imaging assay.** J774 cells in 96-well dishes were infected with GFP-expressing H37Rv at an MOI of 1∶1. Cells were then treated with rifampin at the indicated concentrations. Day 3 after infection, cells were fixed, stained with DAPI, and imaged. (A) Nuclei of macrophages are in blue; GFP-expressing *M. tuberculosis* are in green. (B) GFP channel only. For each image set image contrast was adjusted equally for every image to promote print quality. Images were not adjusted prior to analysis.
(PDF)

Figure S2 **Percent representation of compound categories in input library.**
(PDF)

Figure S3 **Fluoxetine re-testing in J774 macrophages using the image analysis assay.** J774 cells were seeded into 96-well plates at ∼3000 cells/well and allowed to adhere overnight. They were then infected with H37Rv-GFP at an MOI of 1∶1. After 4 hours of phagocytosis, cells were washed, and fluoxetine at the indicated concentrations or DMSO control was added to wells. Day 3 after infection, cells were fixed and stained with DAPI. The imaging pipeline described in detail in Methods S1 was used to measure normalized integrated GFP intensity (A) and quantify DAPI-stained nuclei compared to DMSO control for each condition (B).
(PDF)

Figure S4 **Gefitinib re-testing in J774 macrophages using the image analysis assay.** J774 cells were seeded into 96-well plates at ∼3000 cells/well and allowed to adhere overnight. They were then infected with H37Rv-GFP at an MOI of 1∶1. After 4 hours of phagocytosis, cells were washed, and gefitinib at the indicated concentrations or DMSO control was added to wells. Day 3 after infection, cells were fixed and stained with DAPI. The imaging pipeline described in detail in Methods S1 was used to measure normalized integrated GFP intensity (A) and quantify DAPI-stained nuclei compared to DMSO control for each condition (B).
(PDF)

Figure S5 **GNF2 re-testing in J774 macrophages using the image analysis assay.** J774 cells were seeded into 96-well plates at ∼3000 cells/well and allowed to adhere overnight. They were then infected with H37Rv-GFP at an MOI of 1∶1. After 4 hours of phagocytosis, cells were washed, and GNF2 at the indicated concentrations or DMSO control was added to wells. Day 3 after infection, cells were fixed and stained with DAPI. The imaging pipeline described in detail in Methods S1 was used to measure normalized integrated GFP intensity (A) and quantify DAPI-stained nuclei compared to DMSO control for each condition (B).
(PDF)

Figure S6 **AKTi1/2 re-testing in J774 macrophages using the image analysis assay.** J774 cells were seeded into 96-well plates at ∼3000 cells/well and allowed to adhere overnight. They were then infected with H37Rv-GFP at an MOI of 1∶1. After 4 hours of phagocytosis, cells were washed, and AKTi1/2 at the indicated concentrations or DMSO control was added to wells. Day 3 after infection, cells were fixed and stained with DAPI. The imaging pipeline described in detail in Methods S1 was used to measure normalized integrated GFP intensity (A) and quantify DAPI-stained nuclei compared to DMSO control for each condition (B).
(PDF)

Figure S7 **FTT re-testing in J774 macrophages using the image analysis assay.** J774 cells were seeded into 96-well plates at ∼3000 cells/well and allowed to adhere overnight. They were then infected with H37Rv-GFP at an MOI of 1∶1. After 4 hours of phagocytosis, cells were washed, and FTT at the indicated concentrations or DMSO control was added to wells. Day 3 after infection, cells were fixed and stained with DAPI. The imaging pipeline described in detail in Methods S1 was used to measure normalized integrated GFP intensity (A) and quantify DAPI-stained nuclei compared to DMSO control for each condition (B).
(PDF)

Figure S8 **Ritanserin re-testing in J774 macrophages using the image analysis assay.** J774 cells were seeded into 96-well plates at ∼3000 cells/well and allowed to adhere overnight. They were then infected with H37Rv-GFP at an MOI of 1∶1. After 4 hours of phagocytosis, cells were washed, and ritanserin at the indicated concentrations or DMSO control was added to wells. Day 3 after infection, cells were fixed and stained with DAPI. The imaging pipeline described in detail in Methods S1 was used to measure normalized integrated GFP intensity (A) and quantify DAPI-stained nuclei compared to DMSO control for each condition (B).
(PDF)

Figure S9 **Testing selected hits for activity against** ***M. tuberculosis*** **growing in axenic culture.** *M. tuberculosis* was grown to mid-log phase, then diluted back to an OD600 of 0.05. Compounds were added at the concentrations indicated, and the cultures were incubated at 37°C. On days 3, 7, and 14 after inoculation, cells were mixed, and OD600 was recorded. At the tested concentrations, which are the maximum concentrations used in macrophages, no compounds had significant activity against *M. tuberculosis* in axenic culture.
(PDF)

Figure S10 **Targeting protein expression in J774 macrophages using siRNA.** J774 macrophages were transfected with a pool of 5 siRNAs targeting AKT1, AKT2, AKT3, or ABL1 on two consecutive days. Control samples were transfected with a pool of 5 nonspecific siRNAs. 24 h after the second transfection the cells were harvested and split into fresh plates for 24 h at which time lysates were prepared for (A) Western blot analysis. To assess protein levels present in the cells at the time of infection, lysates were prepared at the same time-point after transfection that the cells are infected with *M. tuberculosis*. Samples were blotted with antibodies specific for AKT1, AKT2, AKT3 or ABL1. Blots were stripped and reprobed with α–actin for loading control. (B) To assess efficiency of phagocytosis the cells were infected with wild-type H37Rv cells for a period of 4 h at which time the infected monolayers were washed, lysed, and CFU were enumerated by plating on agar plates.
(PDF)

Figure S11 **Detection of EGFR transcript by PCR in J774 cells.** RNA was isolated from J774 cells. 1 µg of RNA was used as template for cDNA production with (+RT) or without (−RT) addition of reverse transcriptase. The cDNA was used as a template for standard PCR using primers to amplify a small fragment of cDNA crossing a splice junction. A band corresponding to transcript was detected in the +RT but not −RT samples after 25 cycles. Shown are the samples after 35 cycles. For comparison, 20 µl of 50 bp ladder (NEB) were run in lane 1. See Methods S1 for full protocol details including primer sequences.
(PDF)

Figure S12 **CellProfiler identification of bacteria and nuclei.** A. Illumination-corrected DAPI image (nuclei). B. CellProfiler identification of nuclei (green outlines: nuclei, red outlines: excluded shapes based on size criteria). C. Illumination-corrected GFP image (bacteria). D. CellProfiler identification of bacteria (green outlines: bacteria).
(PDF)

Methods S1 **Additional methods including statistics, details of assay development, CellProfiler analysis and EGFR PCR.**
(DOCX)

Table S1 **Compounds included in screened library and complete hit list with composite Z-scores and Monte Carlo bootstrap-determined p-values.**
(XLSX)
