# Supplementary figures and images for: Identification of Host-Targeted Small Molecules That Restrict Intracellular Mycobacterium tuberculosis Growth
Source: PLoS Pathog. 2014 Feb 20;10(2):e1003946. doi: 10.1371/journal.ppat.1003946 (PMC3930586; doi:10.1371/journal.ppat.1003946)

Figure S1A

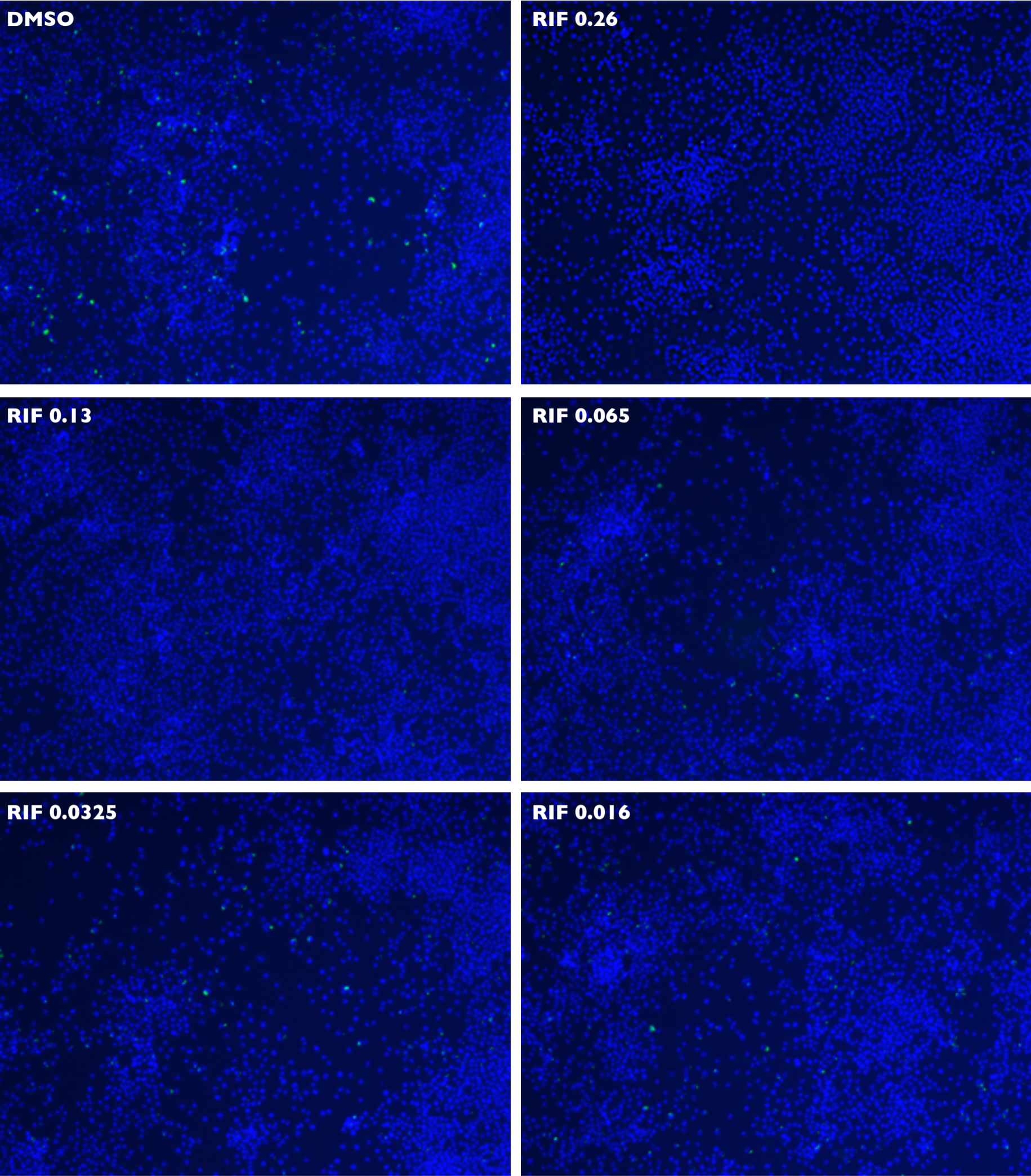

Figure S1B

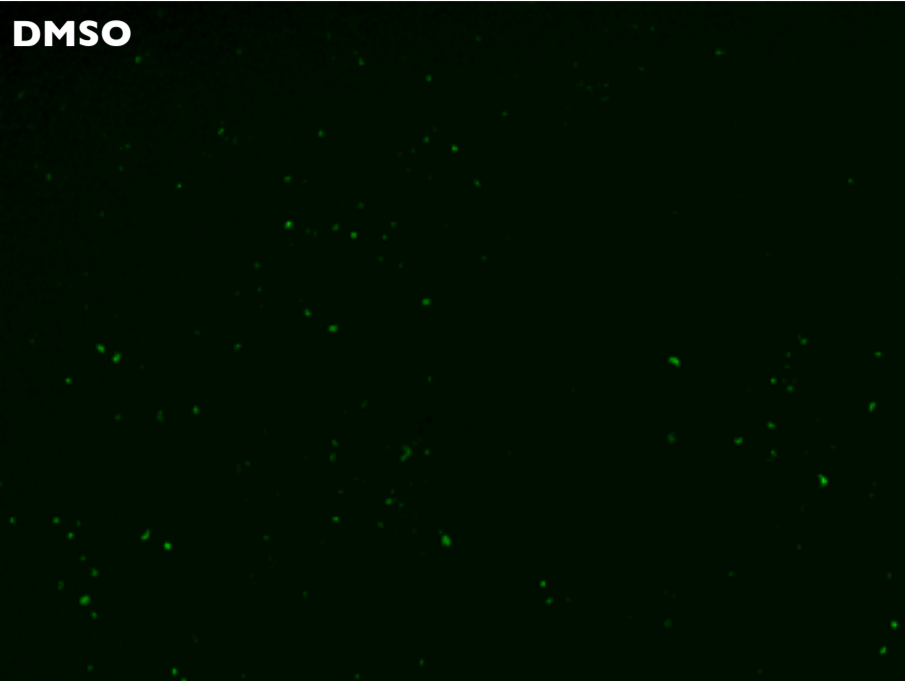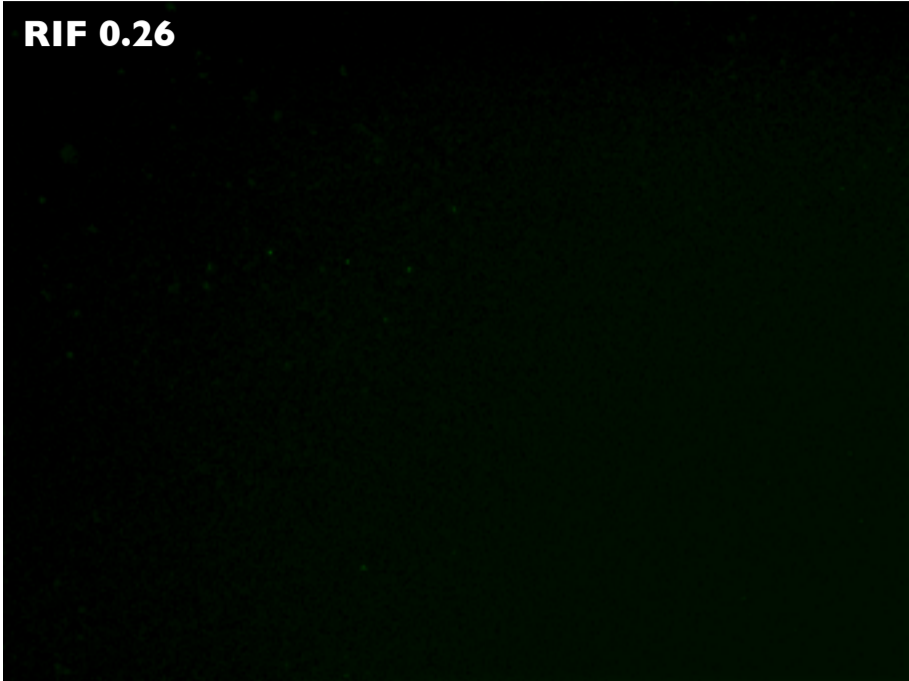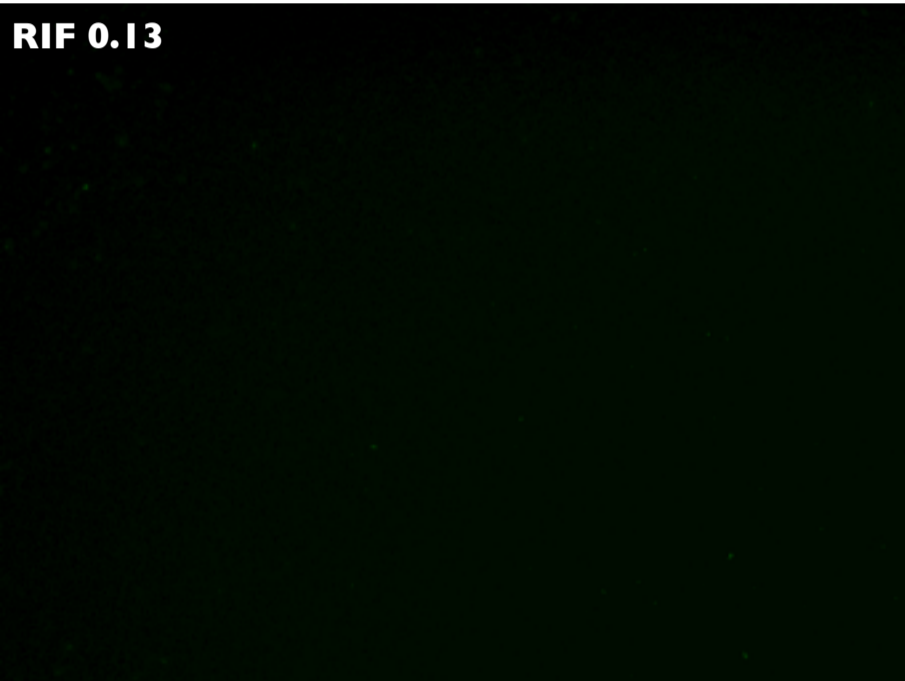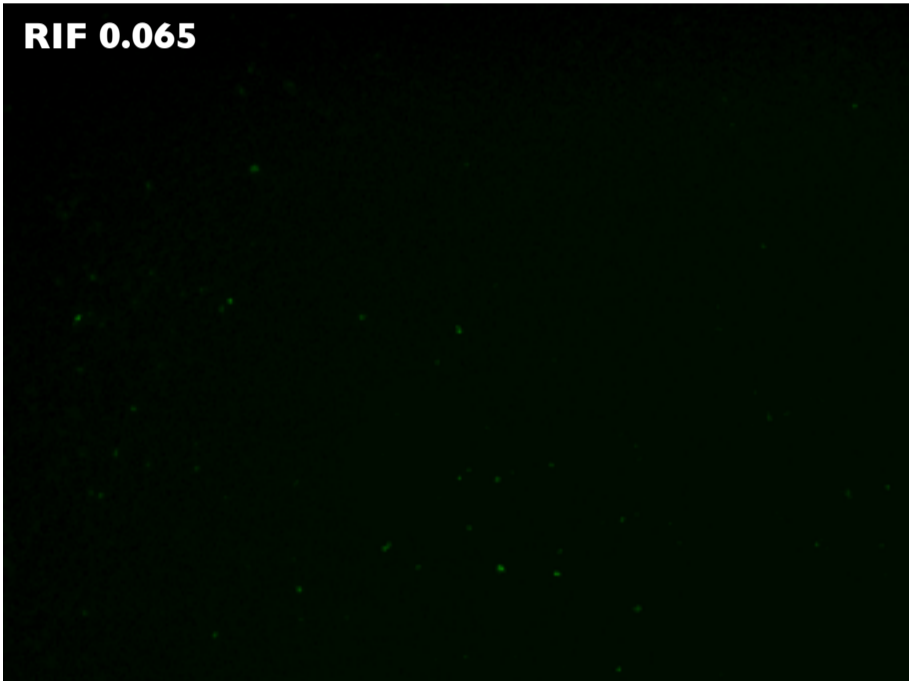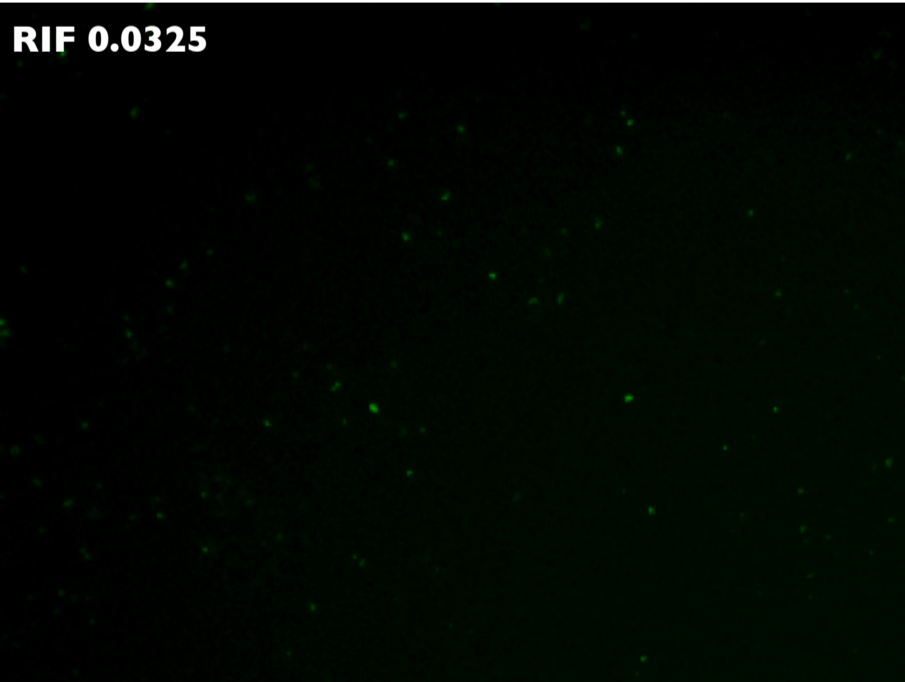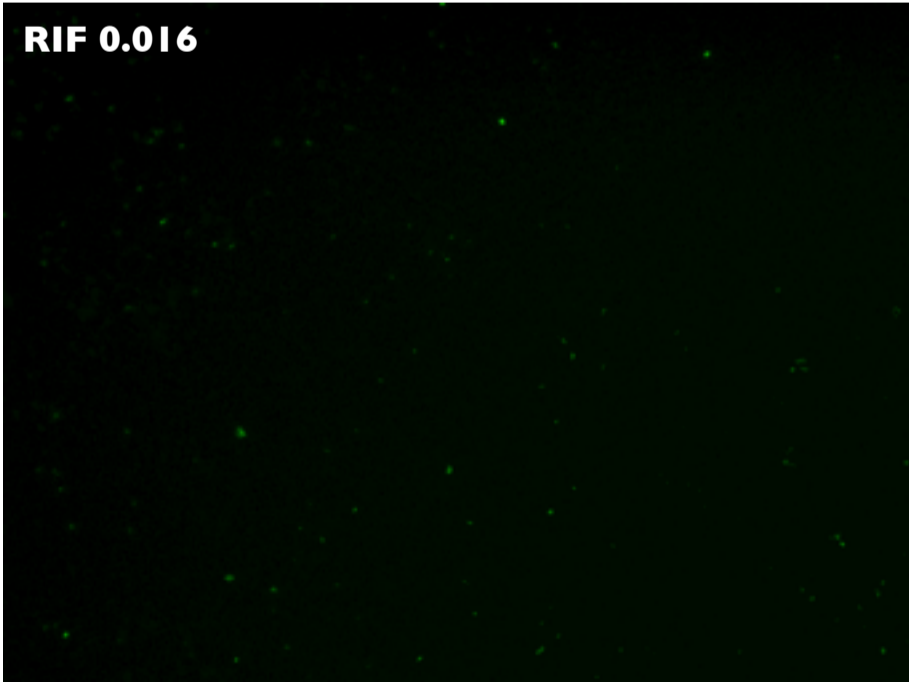

Supplement: Figure S1 — Positive controls for development of high-content imaging assay. J774 cells in 96-well dishes were infected with GFP-expressing H37Rv at an MOI of 1∶1. Cells were then treated with rifampin at the indicated concentrations. Day 3 after infection, cells were fixed, stained with DAPI, and imaged. (A) Nuclei of macrophages are in blue; GFP-expressing M. tuberculosis are in green. (B) GFP channel only. For each image set image contrast was adjusted equally for every image to promote print quality. Images were not adjusted prior to analysis. (PDF) [file ppat.1003946.s001.pdf]

Figure S2

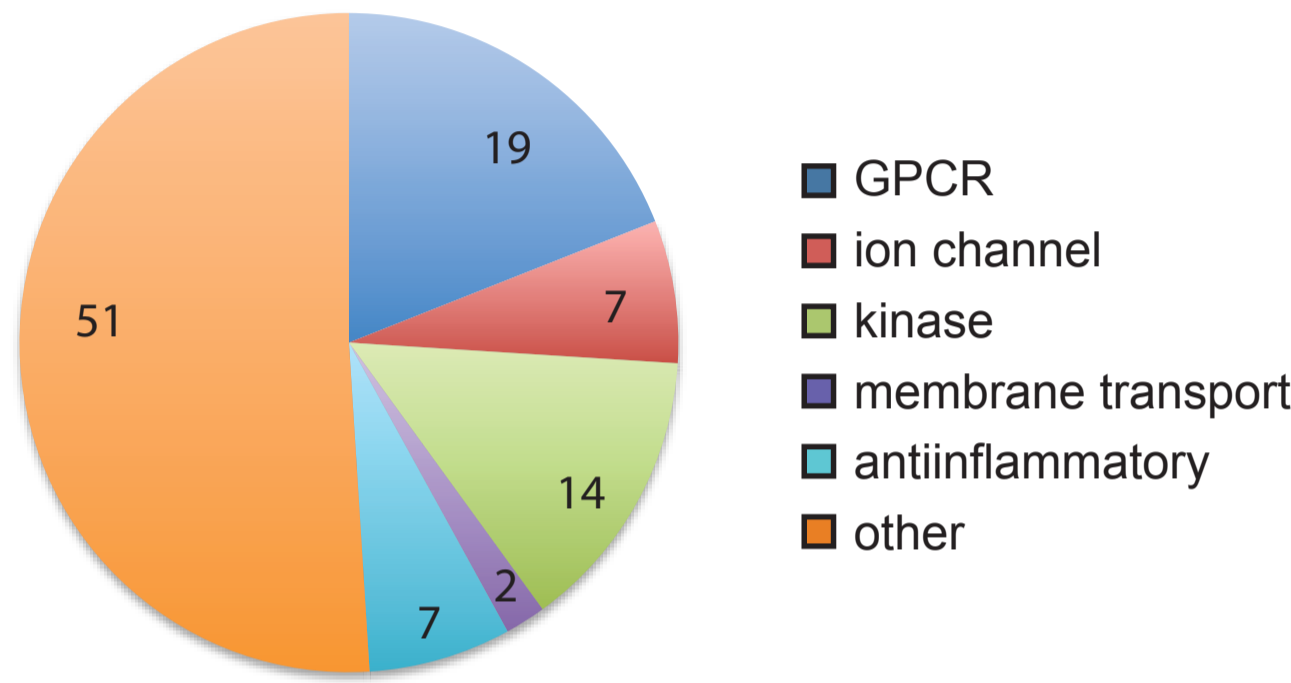

Supplement: Figure S2 — Percent representation of compound categories in input library. (PDF) [file ppat.1003946.s002.pdf]

Figure S3

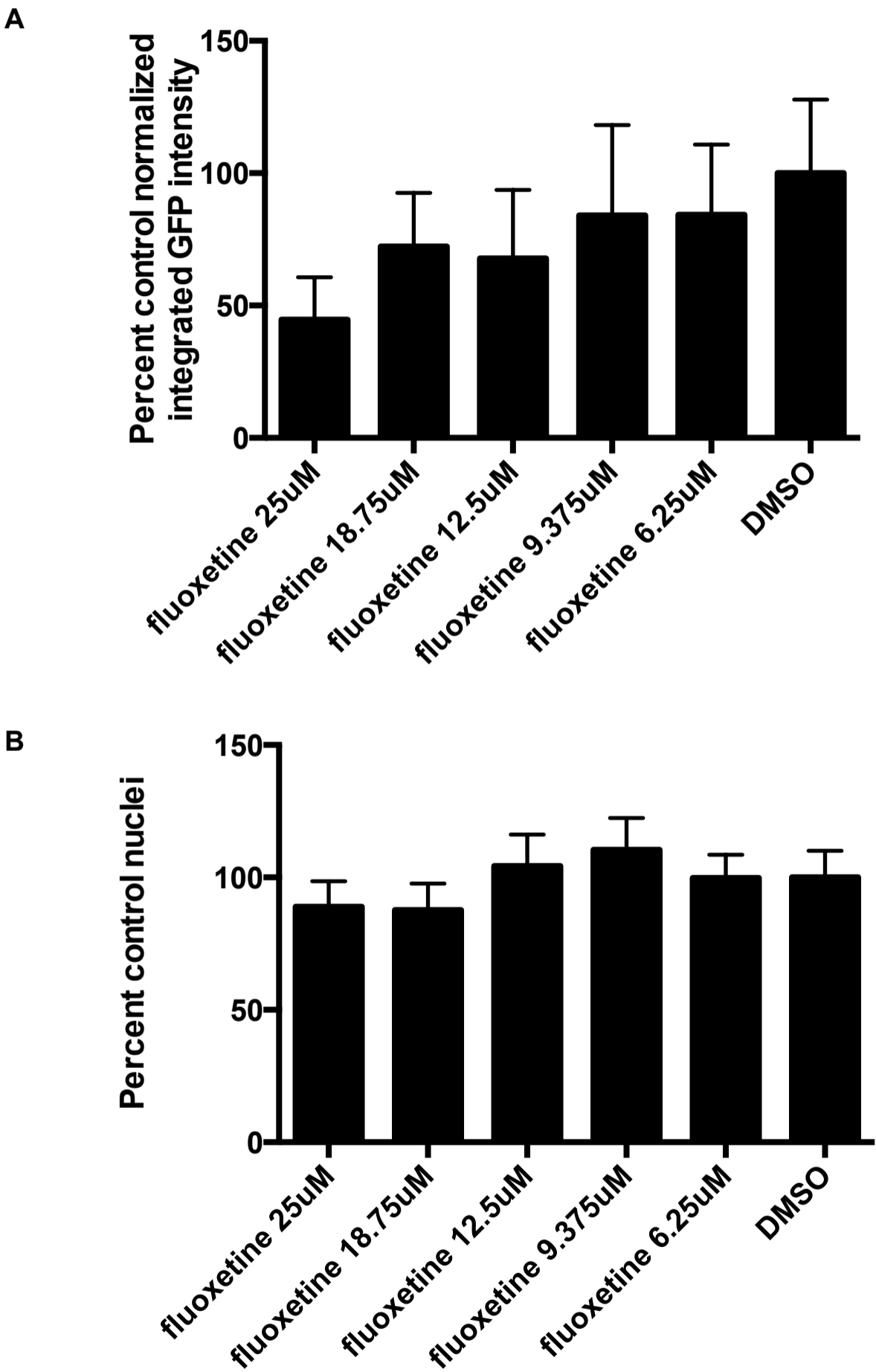

Supplement: Figure S3 — Fluoxetine re-testing in J774 macrophages using the image analysis assay. J774 cells were seeded into 96-well plates at ∼3000 cells/well and allowed to adhere overnight. They were then infected with H37Rv-GFP at an MOI of 1∶1. After 4 hours of phagocytosis, cells were washed, and fluoxetine at the indicated concentrations or DMSO control was added to wells. Day 3 after infection, cells were fixed and stained with DAPI. The imaging pipeline described in detail in Methods S1 was used to measure normalized integrated GFP intensity (A) and quantify DAPI-stained nuclei compared to DMSO control for each condition (B). (PDF) [file ppat.1003946.s003.pdf]

Figure S4

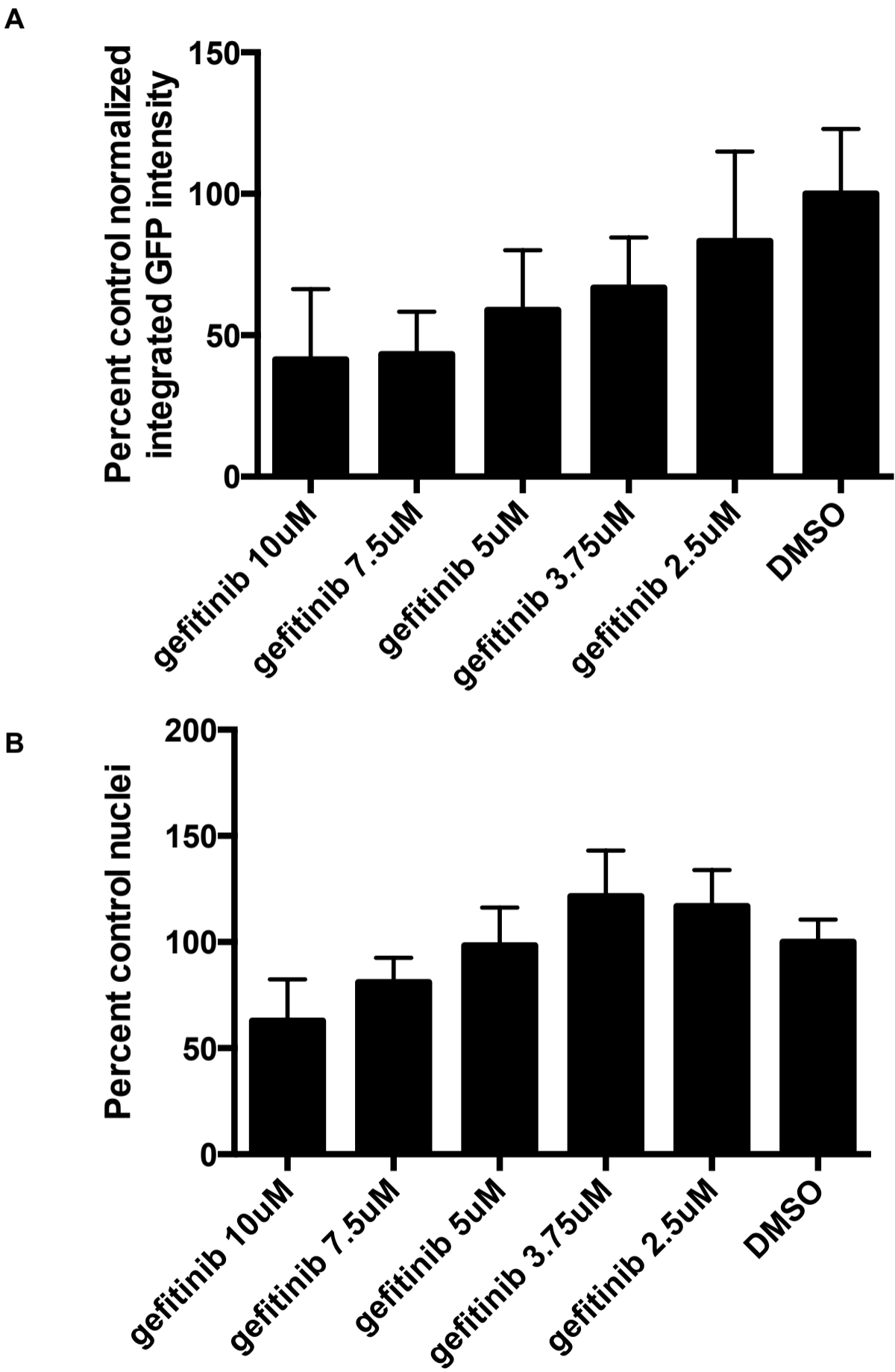

Supplement: Figure S4 — Gefitinib re-testing in J774 macrophages using the image analysis assay. J774 cells were seeded into 96-well plates at ∼3000 cells/well and allowed to adhere overnight. They were then infected with H37Rv-GFP at an MOI of 1∶1. After 4 hours of phagocytosis, cells were washed, and gefitinib at the indicated concentrations or DMSO control was added to wells. Day 3 after infection, cells were fixed and stained with DAPI. The imaging pipeline described in detail in Methods S1 was used to measure normalized integrated GFP intensity (A) and quantify DAPI-stained nuclei compared to DMSO control for each condition (B). (PDF) [file ppat.1003946.s004.pdf]

Figure S5

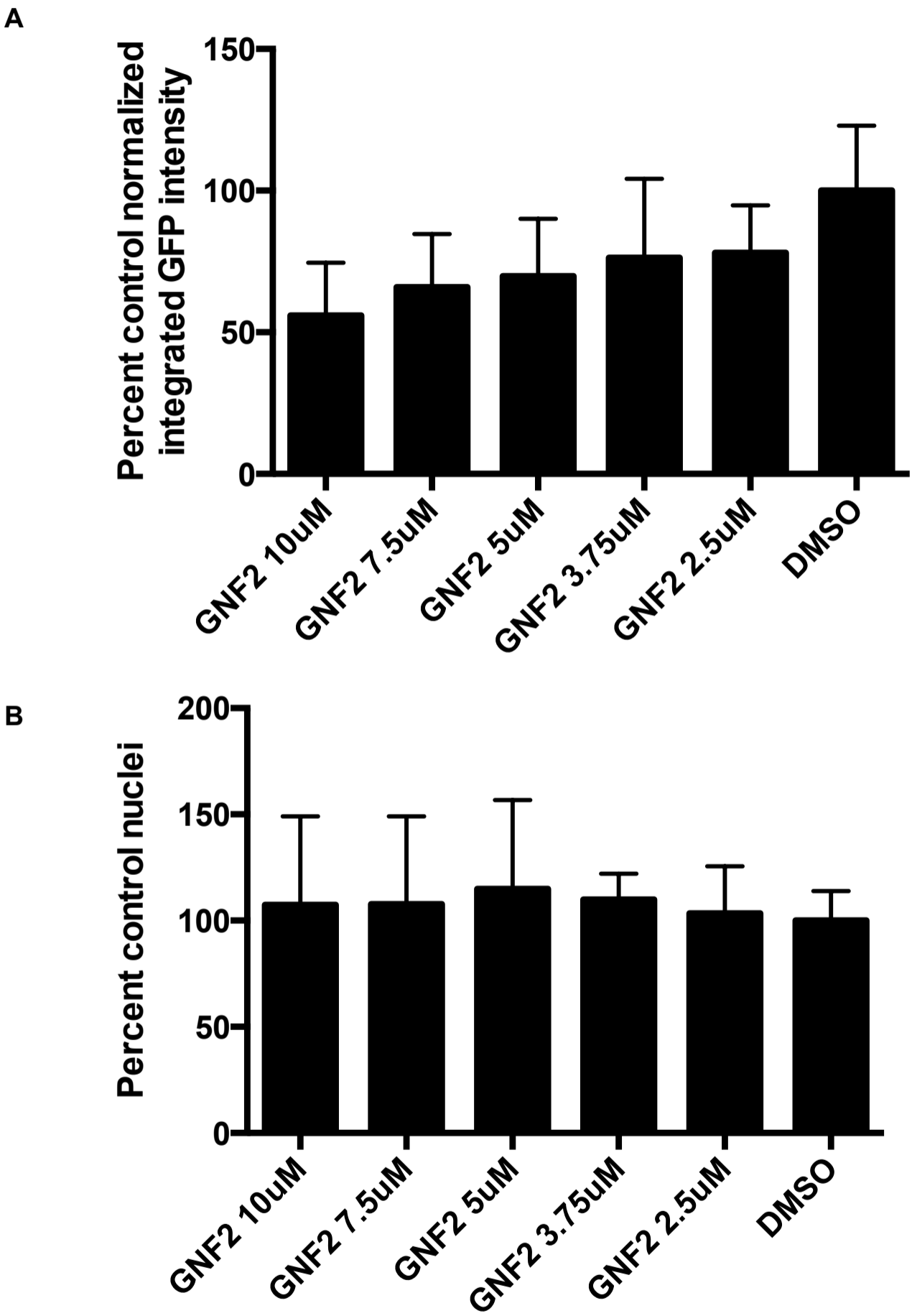

Supplement: Figure S5 — GNF2 re-testing in J774 macrophages using the image analysis assay. J774 cells were seeded into 96-well plates at ∼3000 cells/well and allowed to adhere overnight. They were then infected with H37Rv-GFP at an MOI of 1∶1. After 4 hours of phagocytosis, cells were washed, and GNF2 at the indicated concentrations or DMSO control was added to wells. Day 3 after infection, cells were fixed and stained with DAPI. The imaging pipeline described in detail in Methods S1 was used to measure normalized integrated GFP intensity (A) and quantify DAPI-stained nuclei compared to DMSO control for each condition (B). (PDF) [file ppat.1003946.s005.pdf]

Figure S6

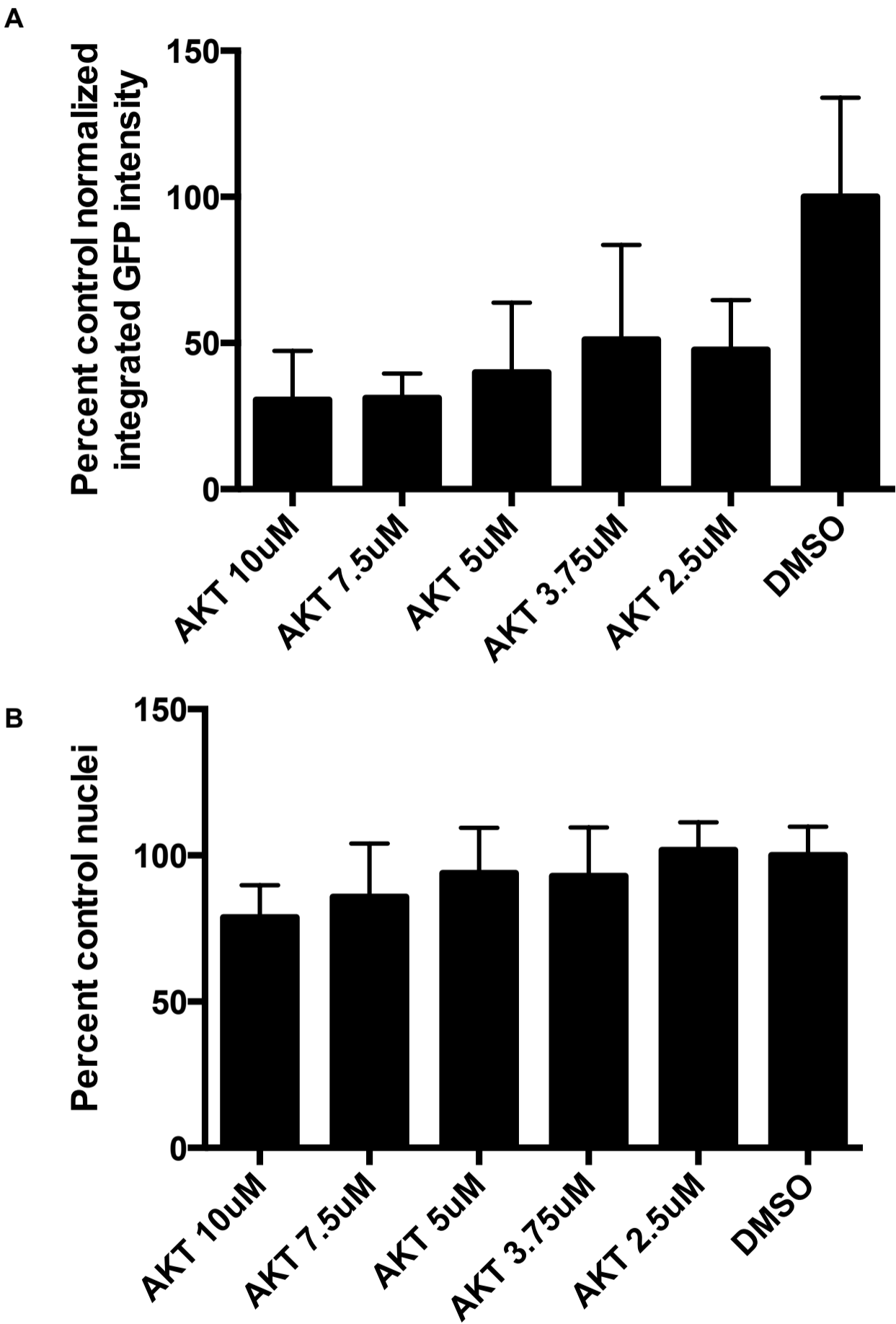

Supplement: Figure S6 — AKTi1/2 re-testing in J774 macrophages using the image analysis assay. J774 cells were seeded into 96-well plates at ∼3000 cells/well and allowed to adhere overnight. They were then infected with H37Rv-GFP at an MOI of 1∶1. After 4 hours of phagocytosis, cells were washed, and AKTi1/2 at the indicated concentrations or DMSO control was added to wells. Day 3 after infection, cells were fixed and stained with DAPI. The imaging pipeline described in detail in Methods S1 was used to measure normalized integrated GFP intensity (A) and quantify DAPI-stained nuclei compared to DMSO control for each condition (B). (PDF) [file ppat.1003946.s006.pdf]

Figure S7

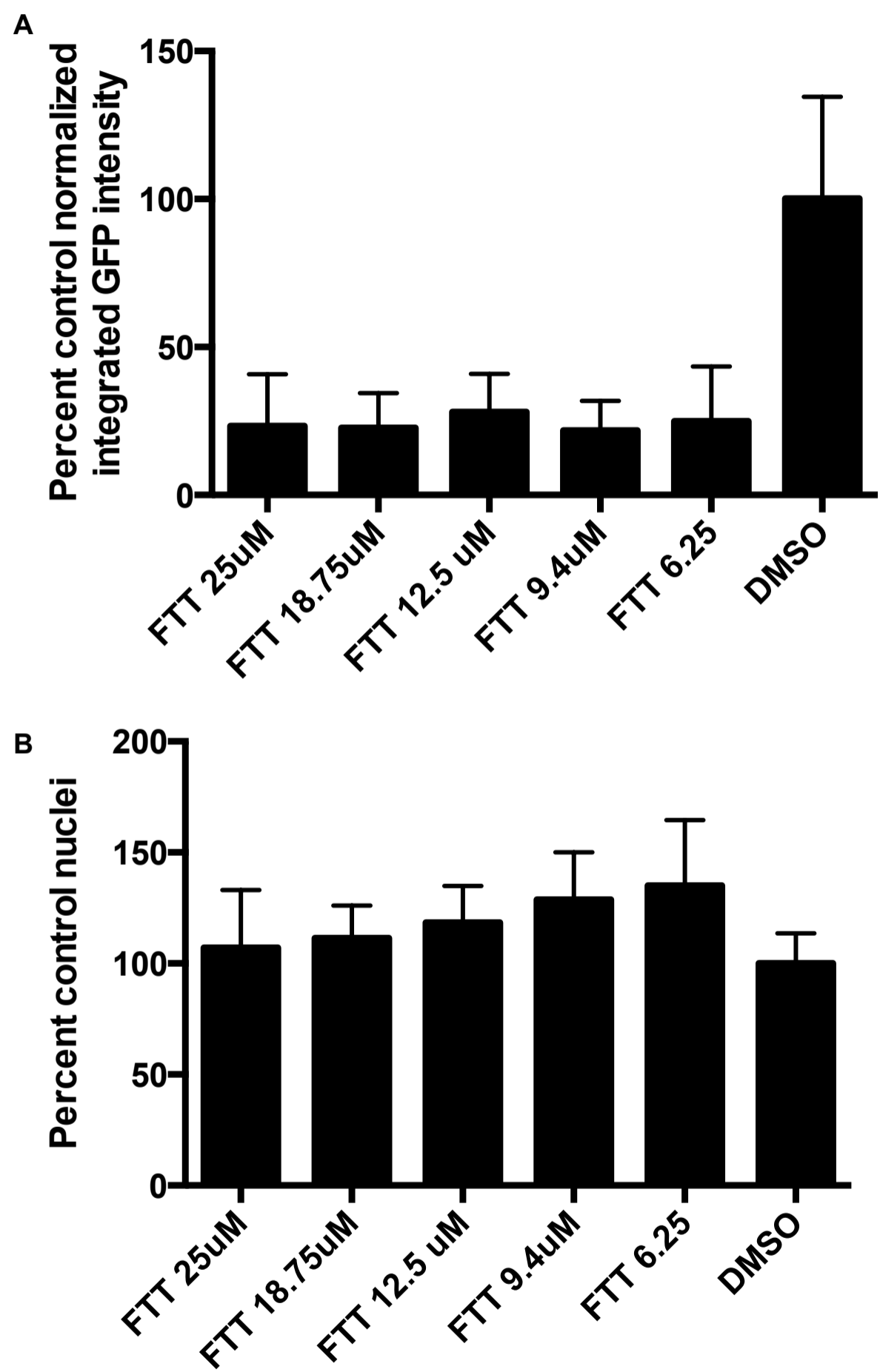

Supplement: Figure S7 — FTT re-testing in J774 macrophages using the image analysis assay. J774 cells were seeded into 96-well plates at ∼3000 cells/well and allowed to adhere overnight. They were then infected with H37Rv-GFP at an MOI of 1∶1. After 4 hours of phagocytosis, cells were washed, and FTT at the indicated concentrations or DMSO control was added to wells. Day 3 after infection, cells were fixed and stained with DAPI. The imaging pipeline described in detail in Methods S1 was used to measure normalized integrated GFP intensity (A) and quantify DAPI-stained nuclei compared to DMSO control for each condition (B). (PDF) [file ppat.1003946.s007.pdf]

Figure S8

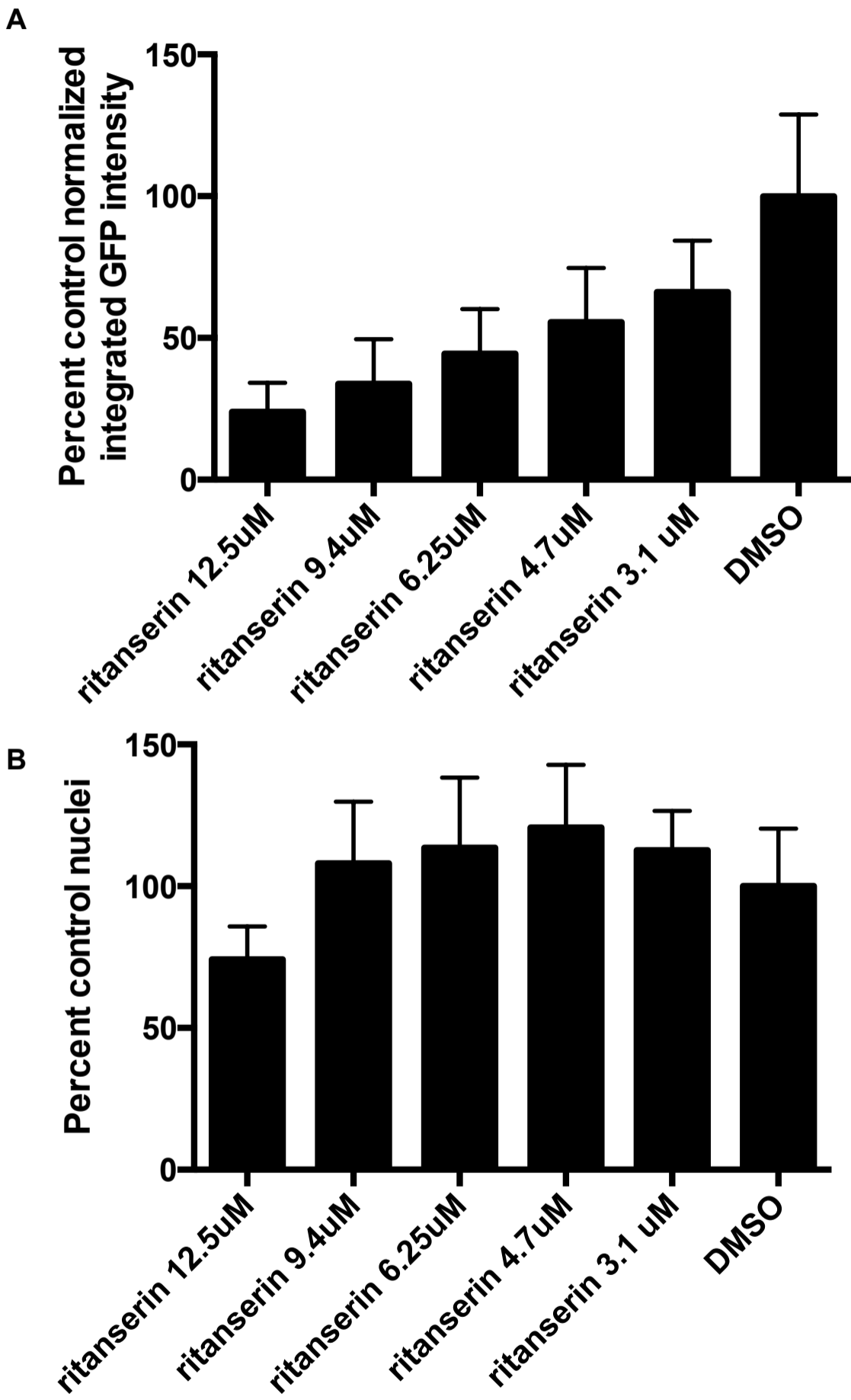

Supplement: Figure S8 — Ritanserin re-testing in J774 macrophages using the image analysis assay. J774 cells were seeded into 96-well plates at ∼3000 cells/well and allowed to adhere overnight. They were then infected with H37Rv-GFP at an MOI of 1∶1. After 4 hours of phagocytosis, cells were washed, and ritanserin at the indicated concentrations or DMSO control was added to wells. Day 3 after infection, cells were fixed and stained with DAPI. The imaging pipeline described in detail in Methods S1 was used to measure normalized integrated GFP intensity (A) and quantify DAPI-stained nuclei compared to DMSO control for each condition (B). (PDF) [file ppat.1003946.s008.pdf]

### Figure S9

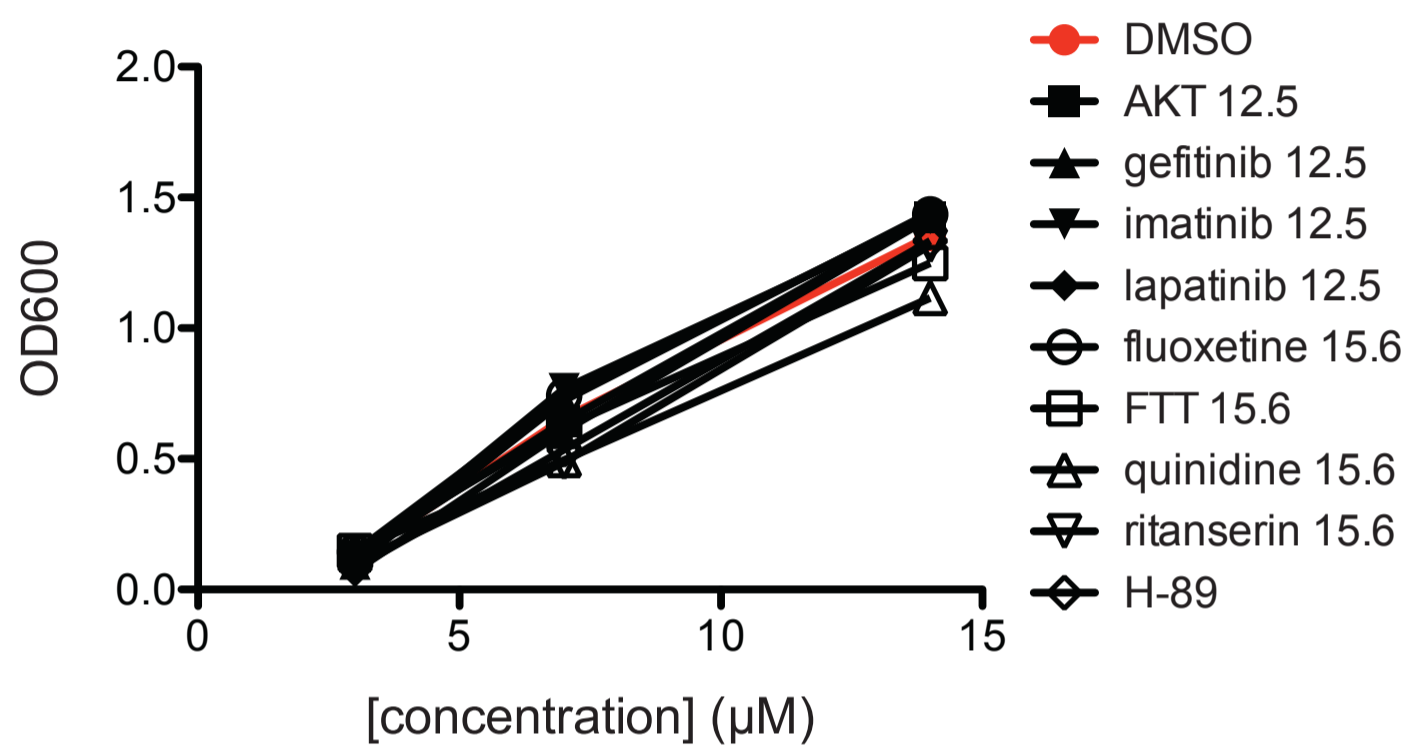

Supplement: Figure S9 — Testing selected hits for activity against M. tuberculosis growing in axenic culture. M. tuberculosis was grown to mid-log phase, then diluted back to an OD600 of 0.05. Compounds were added at the concentrations indicated, and the cultures were incubated at 37°C. On days 3, 7, and 14 after inoculation, cells were mixed, and OD600 was recorded. At the tested concentrations, which are the maximum concentrations used in macrophages, no compounds had significant activity against M. tuberculosis in axenic culture. (PDF) [file ppat.1003946.s009.pdf]

Figure S10

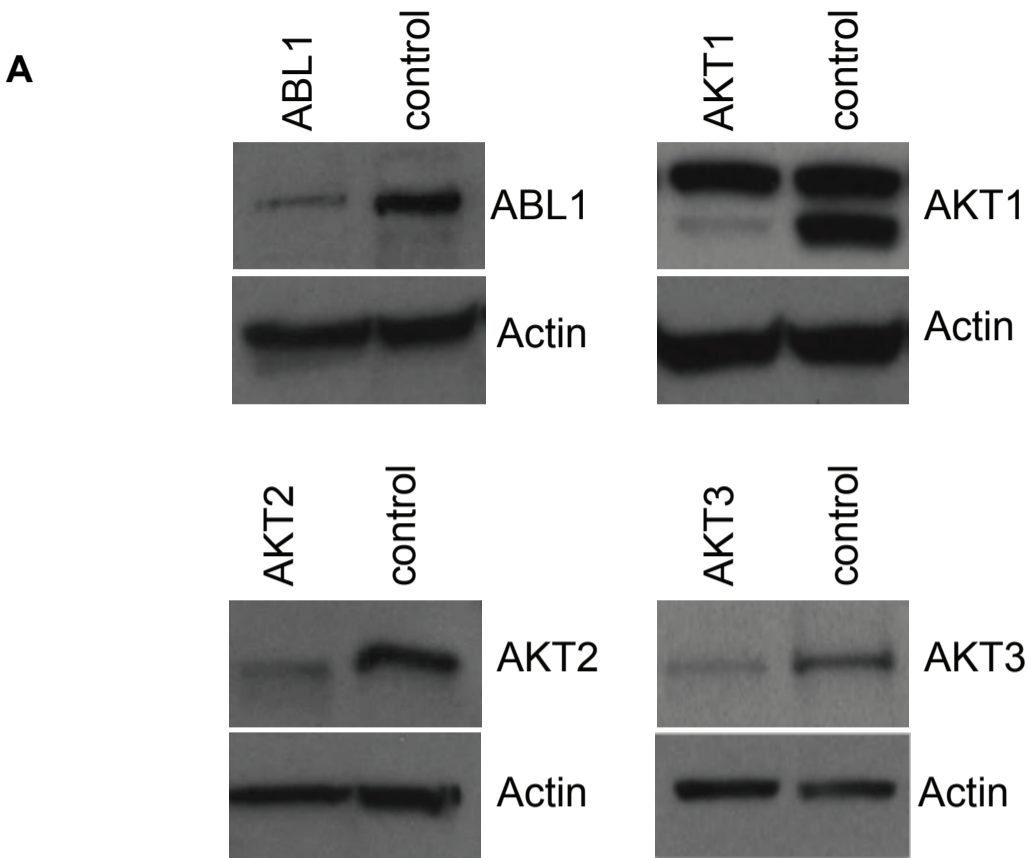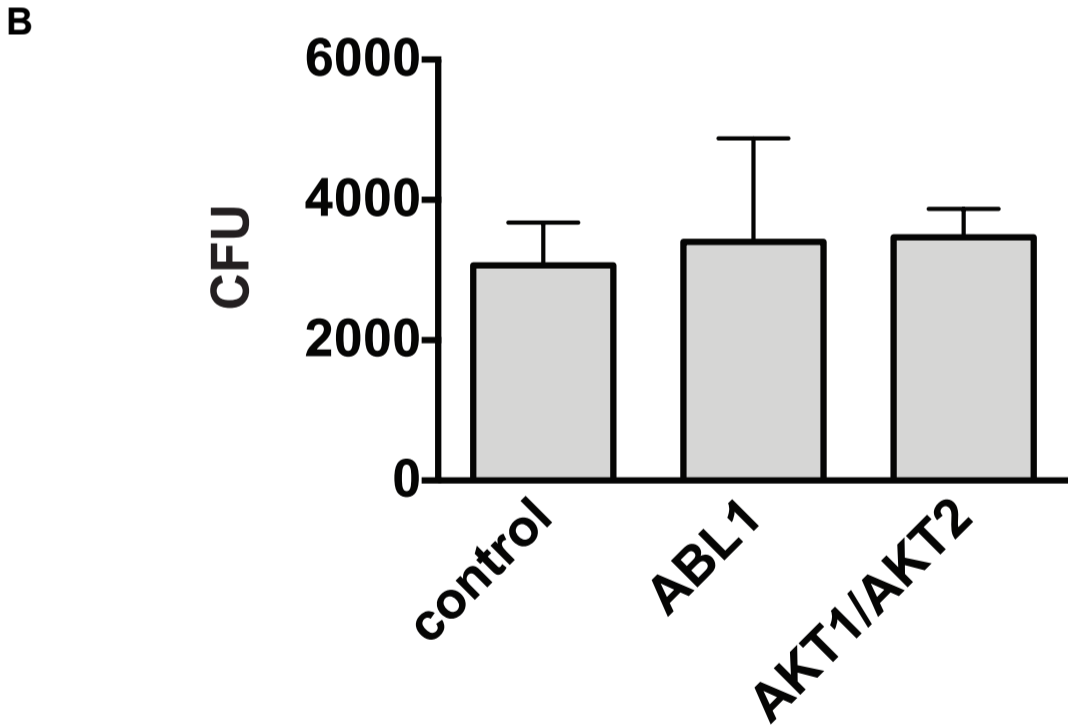

Supplement: Figure S10 — Targeting protein expression in J774 macrophages using siRNA. J774 macrophages were transfected with a pool of 5 siRNAs targeting AKT1, AKT2, AKT3, or ABL1 on two consecutive days. Control samples were transfected with a pool of 5 nonspecific siRNAs. 24 h after the second transfection the cells were harvested and split into fresh plates for 24 h at which time lysates were prepared for (A) Western blot analysis. To assess protein levels present in the cells at the time of infection, lysates were prepared at the same time-point after transfection that the cells are infected with M. tuberculosis. Samples were blotted with antibodies specific for AKT1, AKT2, AKT3 or ABL1. Blots were stripped and reprobed with α–actin for loading control. (B) To assess efficiency of phagocytosis the cells were infected with wild-type H37Rv cells for a period of 4 h at which time the infected monolayers were washed, lysed, and CFU were enumerated by plating on agar plates. (PDF) [file ppat.1003946.s010.pdf]

Figure S11

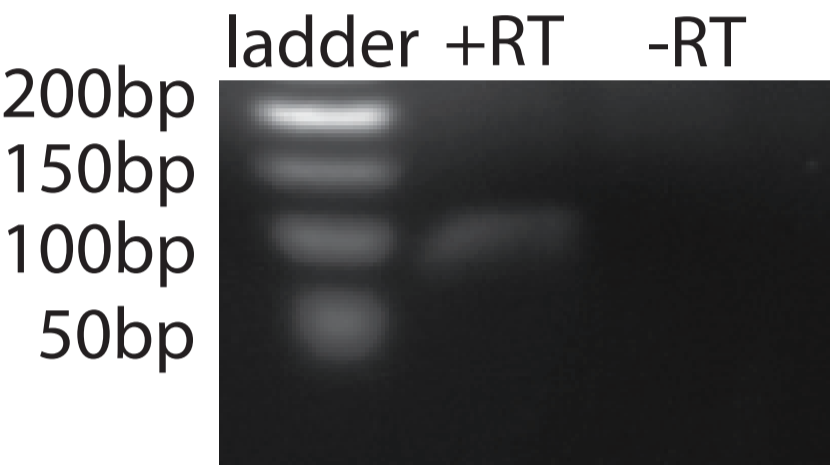

Supplement: Figure S11 — Detection of EGFR transcript by PCR in J774 cells. RNA was isolated from J774 cells. 1 µg of RNA was used as template for cDNA production with (+RT) or without (−RT) addition of reverse transcriptase. The cDNA was used as a template for standard PCR using primers to amplify a small fragment of cDNA crossing a splice junction. A band corresponding to transcript was detected in the +RT but not −RT samples after 25 cycles. Shown are the samples after 35 cycles. For comparison, 20 µl of 50 bp ladder (NEB) were run in lane 1. See Methods S1 for full protocol details including primer sequences. (PDF) [file ppat.1003946.s011.pdf]

Figure S12

A

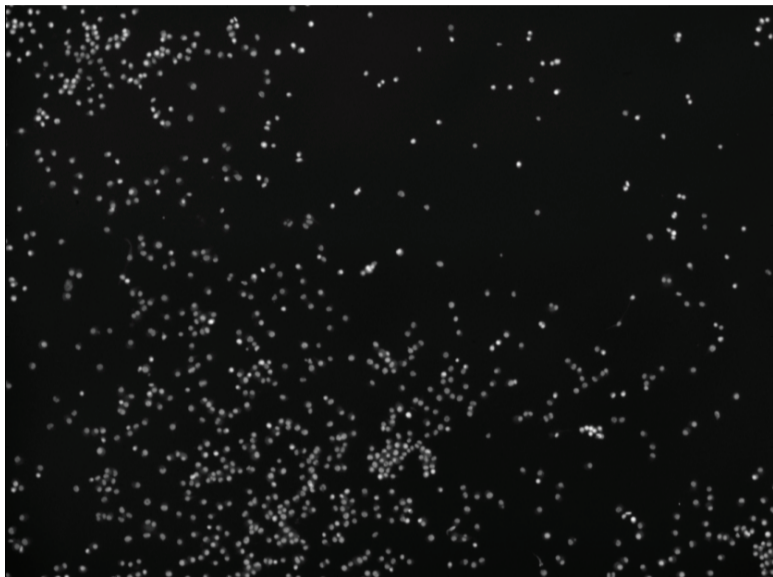

B

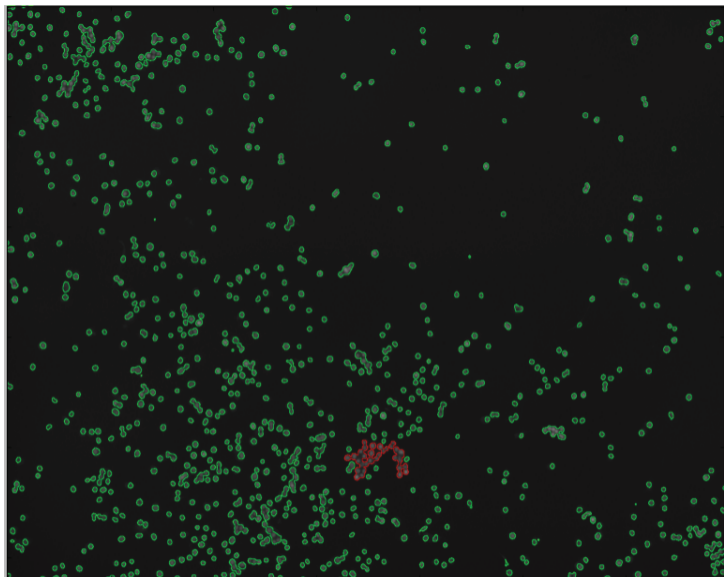

C

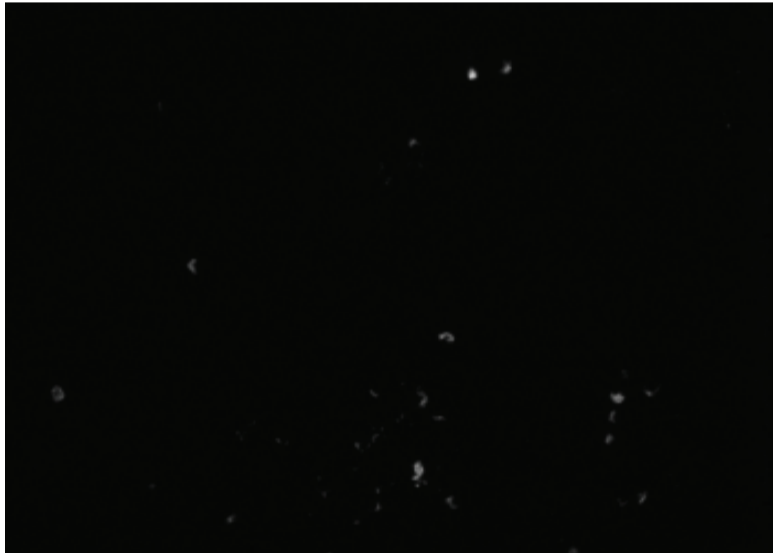

D

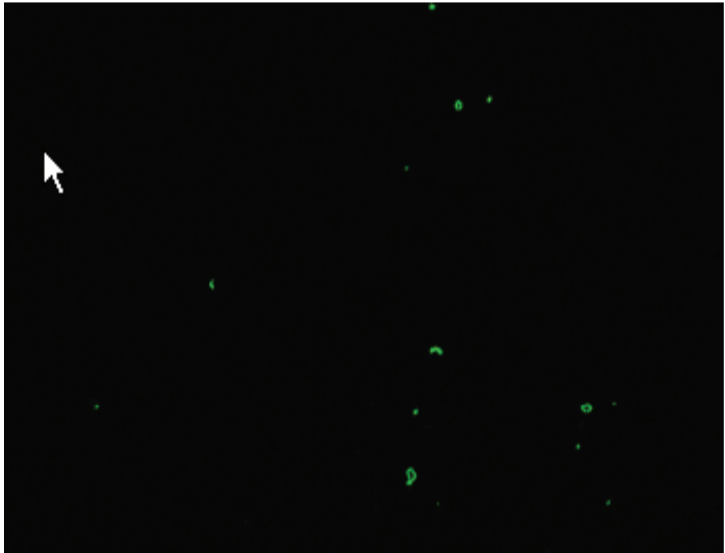

Supplement: Figure S12 — CellProfiler identification of bacteria and nuclei. A. Illumination-corrected DAPI image (nuclei). B. CellProfiler identification of nuclei (green outlines: nuclei, red outlines: excluded shapes based on size criteria). C. Illumination-corrected GFP image (bacteria). D. CellProfiler identification of bacteria (green outlines: bacteria). (PDF) [file ppat.1003946.s012.pdf]
