# Supplementary material for: Identification of Host-Targeted Small Molecules That Restrict Intracellular Mycobacterium tuberculosis Growth
Source: PLoS Pathog. 2014 Feb 20;10(2):e1003946. doi: 10.1371/journal.ppat.1003946 (PMC3930586; doi:10.1371/journal.ppat.1003946)
Supplement: Methods S1 — Additional methods including statistics, details of assay development, CellProfiler analysis and EGFR PCR. (DOCX) [file ppat.1003946.s013.docx]

**Statistics**

**Bootstrap Monte Carlo**

We used the Monte Carlo method of random sampling to generate a distribution for DMSO negative controls. Through the text below, the ‘readout’ for a treatment refers to the average value of the duplicate pair for that treatment. In order to generate an appropriate null distribution for the readouts, *averages of pairs* of randomly sampled DMSO wells (from N=540 wells) were used to generate a null distribution. We determined a cutoff *v* in the resulting distribution by selecting acceptable false positive rates of 0.13% or 0.013%. To determine the corresponding false negative rate with respect to the rifampicin positive control, we similarly generated a distribution for the positive control, i.e., averages of pairs of randomly sampled rifampicin wells (from N=32 wells per dose) were used. The false positive rate was given by the fraction of the positive control readouts that fell *above* the threshold *v*. Using this method, at 50% inhibition we observed a false negative rate of 0.195%, indicating that our assay was robust to detect even modest levels of inhibition. The performance of the assay at greater levels of inhibition was even more robust.

| **[Rifampicin]uM** | **% inhibition** | **% false negative rate**  **at 0.13% false positives** | **% false negative rate**  **at 0.013% false positives** |
| --- | --- | --- | --- |
| 0.0163 | 50% | 0.195 | 4.19 |
| 0.0325 | 65% | 0.165 | 0.78 |
| 0.065 | 85% | < 1e-4 | <1e-4 |
| 0.13 | 95% | < 1e-4 | <1e-4 |
| 0.26 | 98% | < 1e-4 | <1e-4 |

**p-value calculation using Bootstrap Monte Carlo**

For a given compound, the p-value was determined by calculating the fraction of samples from the null distribution (generated above) that were below the readout for that compound. In specific, the p-value for a compound is given by

**

where n = the total number of samples in the null distribution and r = the number of samples from the null distribution with a value below the readout for that compound.

**z-score calculation**

For each replicate in the screen a z-score was calculated using the following formula:

where x = the normalized integrated intensity, μ = the mean of the DMSO controls, and σ = the standard deviation of the DMSO controls. To calculate the composite z-score each individual z-score (z1, z2) was combined according to the following formulas:

For z1 < 0:

For z1 > 0:

Composite z-score:

**p-value calculation**

GraphPad Prism was used to calculate exact p-values using a two-tailed Mann-Whitney U rank sum test.

**Assay Development**

We sought to develop an assay that is sufficiently sensitive to detect modest growth inhibition, able to easily identify (and thus eliminate) compounds with significant macrophage toxicity, and amenable to automation to allow for the rapid testing of thousands of compounds. The use of a bacterial luciferase reporter endogenously expressing both enzyme and substrate in *M. tuberculosis* was one option for reading out bacterial number; however, at the time of assay development, we were unable to obtain a strain of *M. tuberculosis* expressing the bacterial *luxCDABE* operon that was not growth-attenuated. We attempted to utilize firefly luciferase; however, we found that the thick mycobacterial cell wall restricted access of the substrate luciferin, and thus bead-beating was required for use of this reporter. As this was not adaptable to high-throughput screening, we turned to testing of fluorescent reporters. Although we tested a panel of mycobacterial promoters for high-level expression of GFP in H37Rv, we were unable to obtain a strain that provided enough sensitivity for detection using a plate reader. We therefore turned to microscopy, as this method provided sensitivity for detection of bacteria and the ability to simultaneously read out bacterial number and macrophage survival.

We next sought to optimize image acquisition and analysis methods using infection of J774 macrophages. Because intracellular mycobacteria grow in clumps rather than discrete, easily quantifiable spots, we sought image analysis software that would allow for determination of a more nuanced growth phenotype than bacterial count. The open-source software platform CellProfiler allowed simultaneous determination of multiple parameters from our images (see description of analysis pipeline below). These measured features include multiple measures of bacterial and nuclear size, the distance between the nucleus and mycobacteria, and various measures of the intensity and texture of each bacterial clump and each nucleus. Using rifampicin treatment as a positive control, we found that the metric that best represented growth was the GFP pixel intensity integrated across the field and normalized to macrophage number, giving Z’-factors of 0.4-0.5 consistently.

Although we used J774 cells to develop the image analysis pipeline, we next sought to establish the optimal cell type for our screen. Growth of *M. tuberculosis* constitutively expressing GFP (H37Rv-GFP) was monitored in various macrophage primary cells and cell lines including primary murine bone marrow derived macrophages, the mouse macrophage cell lines RAW and J774, and the human monocyte/macrophage cell line THP-1. For THP-1 cells, monocytes were plated into 96-well plates and stimulated with PMA to promote differentiation of monocytes into macrophages. Multiple concentrations of PMA and stimulation time were tested. For all other macrophages, the cells were plated into 96-well plates and allowed to adhere overnight. The following day, macrophages were infected with H37Rv-GFP *M. tuberculosis*, and at various time points after infection the cells were fixed and stained with DAPI to allow for enumeration of macrophages. THP-1 cells allowed for robust growth of *M. tuberculosis*; however, by three days after infection (required for optimal separation of positive and negative controls) there was a significant amount of macrophage cell death. Further, the degree of death observed was not consistent across experiments, which would complicate analysis of a screen carried out across multiple screening days. RAW macrophages supported robust growth of *M. tuberculosis*, but excessive proliferation over the three-day growth period (even when seeded at low densities) complicated our image analysis. Bone-marrow macrophages did not support robust growth of *M. tuberculosis*: even after one week of infection we found bacterial numbers had only increased by ~7x and we were unable to obtain Z’- factors greater than 0 in these cells. In contrast, we were able to obtain a Z’-factor of 0.4-0.5 for J774 macrophages in our screening assay.

We next assessed the dynamics of growth of *M. tuberculosis* in J774 macrophages by examining bacterial replication across forty 96-well plates using the drug rifampicin as a control. In each of three replicates carried out on different days, we screened 40 identical 96-well plates, each of which contained DMSO control wells and a dose response of rifampicin. We found that bacterial growth did not follow a Gaussian distribution. We tested whether this was due to a technical aspect of the screen, considering the position of a well on a 96-well plate (including edge effects), position of the plate in the incubator, infection time, order of reading plates, and number of plates assayed simultaneously. None of these technical factors contributed to the non-Gaussian nature of our data. We therefore relied on a Bootstrap Monte Carlo analysis, a statistical methods that does not assume a normal distribution, for assay development. Using a dose response of rifampicin to model varying degrees of growth inhibition, we demonstrated that the assay was able to distinguish even modest levels of growth inhibition (Figure 1, Figure S1). Using a Bootstrap Monte Carlo analysis, we were able to consistently distinguish 50% inhibition of growth with a predicted false negative rate of 0.2% at a false positive rate of 0.13%. Further, we rigorously tested variability using the coefficient of variance (standard deviation divided by the mean) for well-to-well variability (19%), plate-to-plate variability (9%), and day-to-day variability (15%) in the DMSO control wells. Taken together, this analysis demonstrates that the assay is robust for screening.

**CellProfiler analysis**

For image acquisition, we collected four sites per well of the 96-well plate (average of ~7000 macrophages) using an ImageXpress automated microscope (Molecular Devices) with a 4x objective. The image analysis workflow we developed using CellProfiler begins with running a pipeline for correcting fluorescent images for spatial illumination heterogeneities introduced by the optical path in the microscope. Because the amount of mycobacteria is proportional to the fluorescence intensity of GFP recorded in the image, this is a critically important step for accurate measurements of mycobacteria per macrophage. To obtain an accurate estimation of illumination anomalies, this pipeline creates an illumination correction function, estimated as a smoothed per-channel average of the image intensities for a given set of plates imaged on the same day. A second analysis pipeline then corrects each fluorescent image by per-pixel division with the corresponding illumination correction function^1^. After the images are corrected, the pipeline then identifies fluorescent debris in the images under the assumption that such artifacts are larger and brighter than the actual cells. Regions of the image containing debris are then masked from the image to exclude these features from consideration, allowing for more accurate counting of both bacteria and macrophages. Next, a focus score is calculated for each image, which allows us to exclude out-of-focus images from subsequent analysis. From the remaining corrected images, the pipeline then identifies the nuclei using a three-step approach: automatically threshold the DNA-stained (DAPI) foreground from the image background, determine the nuclei boundaries in the foreground regions and separate touching nuclei from their neighbors. The pipeline then identifies the mycobacteria in each GFP image following a similar three-step process except that the detection settings are optimized for the smaller mycobacterial features. For analysis of each set of images, the parameters for defining nuclei and bacteria are run on a small subset of images and verified by eye to confirm accuracy before the entire set is run (see Figure S12). The final step in the analysis pipeline is to measure multiple features of the bacteria and nuclei in order to score the growth of *M. tuberculosis* in macrophage cells. To assign hits, the GFP intensity from the identified bacteria is integrated across each image and is then normalized to the number of macrophage nuclei counted in the image to produce the final readout. Both of the pipelines used in the screen can be found at <http://cellprofiler.org/published_pipelines.html>.

**EGFR PCR**

RNA was extracted from J774 macrophages using the RNeasy kit (Qiagen) and Qiashredder columns (Qiagen). 1μg of RNA was used to synthesize cDNA using the SuperScript III kit (Invitrogen). A control sample with water in place of reverse transcriptase (RT) was made simultaneously. 5μl of cDNA made in the presence or absence of RT was used as template for a PCR reaction run with KOD Xtreme polymerase (Millipore) with the following primer sequences designed to cross a splice junction in EGFR RNA. F primer: AACTGGACTGACCTCCATGC R primer: CGCCAAAGAAAACTGACCAT. The reactions were run for 15, 25, or 35 cycles. A band appeared in the +RT lane after 25 and 35 cycles when 15μl was run out on a 2% agarose gel. Shown are the results for 35 cycles; for comparison 20μl of 50bp ladder (NEB) was run simultaneously.

**Supplementary Reference**

1. Jones TR, Carpenter AE, Sabatini DM, Golland P: Methods for high-content, high-throughput image-based cell screening. In *Proceedings of the Workshop on Microscopic Image Analysis with Applications in Biology held in association with MICCAI06 (Medical Image Computing and Computer-Assisted Intervention)* held in Copenhagen, Denmark, October 5, 2006 Edited by Metaxas DN, Whitaker RT, Rittcher J, Sebastian T. :65-72.
